# The genome of the polar eukaryotic microalga *Coccomyxa subellipsoidea *reveals traits of cold adaptation

**DOI:** 10.1186/gb-2012-13-5-r39

**Published:** 2012-05-25

**Authors:** Guillaume Blanc, Irina Agarkova, Jane Grimwood, Alan Kuo, Andrew Brueggeman, David D Dunigan, James Gurnon, Istvan Ladunga, Erika Lindquist, Susan Lucas, Jasmyn Pangilinan, Thomas Pröschold, Asaf Salamov, Jeremy Schmutz, Donald Weeks, Takashi Yamada, Alexandre Lomsadze, Mark Borodovsky, Jean-Michel Claverie, Igor V Grigoriev, James L Van Etten

**Affiliations:** 1 Structural and Genomic Information Laboratory, UMR7256 CNRS, Aix-Marseille University, Mediterranean Institute of Microbiology (FR3479), Marseille, FR-13385, France; 2 Department of Plant Pathology and Nebraska Center for Virology, University of Nebraska - Lincoln, Lincoln, NE 68583-0722, USA; 3 DOE Joint Genome Institute, Walnut Creek, CA 94598, USA; 4 Department of Biochemistry, University of Nebraska, Lincoln, NE 68588, USA; 5 Department of Applied Ecology, University of Rostock, Department Applied Ecology, Albert-Einstein-Str. 3, D-18059 Rostock, Germany; 6 Department of Molecular Biotechnology, Graduate School of Advanced Sciences of Matter, Hiroshima University, 1-3-1 Kagamiyama, Higashi-Hiroshima 739-8530, Japan; 7 Georgia Tech Center for Bioinformatics and Computational Genomics, Joint Georgia Tech and Emory Wallace H Coulter Department of Biomedical Engineering, Atlanta, GA 30332, USA

## Abstract

**Background:**

Little is known about the mechanisms of adaptation of life to the extreme environmental conditions encountered in polar regions. Here we present the genome sequence of a unicellular green alga from the division chlorophyta, *Coccomyxa subellipsoidea *C-169, which we will hereafter refer to as C-169. This is the first eukaryotic microorganism from a polar environment to have its genome sequenced.

**Results:**

The 48.8 Mb genome contained in 20 chromosomes exhibits significant synteny conservation with the chromosomes of its relatives *Chlorella variabilis *and *Chlamydomonas reinhardtii*. The order of the genes is highly reshuffled within synteny blocks, suggesting that intra-chromosomal rearrangements were more prevalent than inter-chromosomal rearrangements. Remarkably, Zepp retrotransposons occur in clusters of nested elements with strictly one cluster per chromosome probably residing at the centromere. Several protein families overrepresented in *C. subellipsoidae *include proteins involved in lipid metabolism, transporters, cellulose synthases and short alcohol dehydrogenases. Conversely, C-169 lacks proteins that exist in all other sequenced chlorophytes, including components of the glycosyl phosphatidyl inositol anchoring system, pyruvate phosphate dikinase and the photosystem 1 reaction center subunit N (PsaN).

**Conclusions:**

We suggest that some of these gene losses and gains could have contributed to adaptation to low temperatures. Comparison of these genomic features with the adaptive strategies of psychrophilic microbes suggests that prokaryotes and eukaryotes followed comparable evolutionary routes to adapt to cold environments.

## Background

Algae consist of an extremely diverse, polyphyletic group of eukaryotic photosynthetic organisms. To characterize the genetic and metabolic diversity of chlorophytes (eukaryotic green algae) and to better understand how this diversity reflects adaptation to different habitats, we sequenced the trebouxiophyceaen *Coccomyxa subellipsoidea *C-169 NIES 2166. C-169 is a small elongated non-motile unicellular green alga (cell size of approximately 3 to 9 μm; Figure S1A in Additional file 1) isolated in the polar summer of 1959/60 at Marble Point, Antarctica, from dried algal peat [1]. The Antarctic is a particularly harsh environment, with extremely low temperatures (as low as -88°C), frequent and rapid fluctuations from freezing to thawing temperatures, severe winds, low atmospheric humidity, and alternating long periods of sunlight and darkness. C-169 is psychrotolerant with an optimal temperature for growth at around 20°C; in comparison, psychrophiles and psychrototrophs are organisms that have optimal growth temperatures of < 15°C and > 15°C, respectively, and a maximum growth temperature of < 20°C. C-169 was originally classified as *Chlorella vulgaris*, but present sequence data led to re-classification of the alga into the *Coccomyxa *genus with a species name of *C. subellipsoidae *(Supplemental Results in Additional file 2 and Figure S1 in Additional file 1).

*C. subellipsoidea *strains were first isolated in England and Ireland, where they form jelly-like incrustations on mosses and rocks [2,3]. In contrast to its most closely sequenced relative, the trebouxiophyte *Chlorella variabilis *NC64A [4], which is an endosymbiont of paramecia, C-169 is free living. However, the type strain *C. subellipsoidea *SAG 216-13 as well as other isolates in the same species are known to form lichens with subarctic basidiomycetes of the genus *Omphalina *[5]; other *Coccomyxa *spp. are intracellular symbionts of Ginkgo [6] and *Stentors amethystinus *[7] and intracellular parasites of mussels [8]. In the past 20 years C-169 has been used as a model organism in pioneering studies on green algal chromosome architecture. For example, early studies indicated that approximately 1.5% of its genome consists of LINE- and SINE-type retrotransposons [9,10]. Additional studies provided a detailed analysis of the smallest 980 kb chromosome [11,12].

Here we report the gene content, genome organization, and deduced metabolic capacity of C-169 and compare those features to other sequenced chlorophytes. We show that the C-169 gene repertoire encodes enzymatic functions not present in other sequenced green algae that are likely to represent hallmarks of its adaptation to the polar habitat.

## Results and discussion

### Genome structure

The C-169 genome was draft sequenced using the whole genome shotgun Sanger sequencing approach. After sequencing, the C-169 genome was assembled into 29 gap-free scaffolds (12-fold coverage) encompassing 48.8 Mb (Figure S2 in Additional file 1), which is 2.6 Mb (5%) larger than the genome of *C. variabilis *[4]. Alignments of 28,322 ESTs from C-169 indicate that the assembly is 97% complete. Twelve scaffolds represent complete chromosomes with telomeric repeat arrays at both ends. Pulse field gel electrophoresis and Southern hybridization were used to assign the remaining 17 scaffolds to chromosomal bands (Supplemental Results in Additional file 2). This allowed nine scaffolds to be assigned to another four complete chromosomes. The eight remaining scaffolds could not be assigned unambiguiously, because of chromosomes with near identical sizes. These eight scaffolds have a telomeric repeat array at one end; this indicates that they correspond to four additional chromosomes. Thus, sequence assembly and Southern hybridization suggest that the C-169 karyotype consists of 20 chromosomes.

**Figure 1:**
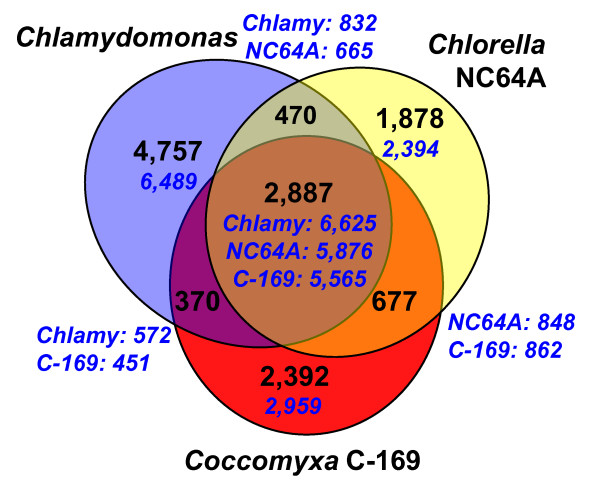
**Venn diagram showing unique and shared gene families between and among three sequenced chlorophyte species (*Coccomyxa subellipsoidea *C-169, *Chlorella variabilis *NC64A, and *Chlamydomonas reinhardtii*)**. Numbers of gene families are indicated in black. Total numbers of genes included in gene families are indicated in blue.

The nuclear genome is 53% GC, with a marked difference between introns (49% GC) and exons (59% GC). However, no long-range variations occur in its GC content as in chlorella and mamiellophycean genomes [4,13]. We predict 9,851 protein-encoding genes (Table 1; Tables S1 and S2, and Supplemental Results in Additional file 2), of which 51% (4,982) are supported by ESTs. Eighty percent of the predicted genes (7,839) have matches in public databases (BLASTP E-value < 1e-5), the majority of which (87%) are most similar to green algae or plant homologs. Although the number of predicted genes is similar in the two trebouxiophytes (Table S3 in Additional file 2), C-169 shares only 6,427 (65%) of its genes with *C. variabilis *(53% (5,232) form reciprocal best hit pairs of putative orthologs) and 5,565 (56%) are shared with *C. reinhardtii *(Figure 1). Like *Chlorella *and *Chlamydomonas *genes (7.3 and 8.3 introns per gene, respectively), C-169 genes have many introns (7.0 introns per gene).

**Table 1:** Genomic features of *C. subellipsoidea *C-169

Characteristic	
Nuclear genome size	48.8 Mb
Chromosome number	20
Number of scaffolds	29
GC (%) genome	53
GC (%) exon	59
GC (%) intron	49
Repeated sequences (%)	7.2
Protein coding gene number	9,851
Mean protein length (amino acids)	425
Gene density (kb/gene)	5.0
Mean exon length	182 bp
Mean intron length	240 bp

About one-third of the mitochondrial genome sequence (20,739/65,497 bp, 31%) and 6% of the chloroplast genome sequence (11,312/175,731 bp) are integrated into the nuclear genome as 385 scattered individual DNA fragments with sizes ranging from 40 to 397 bp (Table S4 in Additional file 2), some containing truncated open reading frames. This phenomenon is more prominent in C-169 than in any sequenced chlorophyte. Both the mitochondrial and chloroplast genomes have GC contents greater than 50% (53.2% for the mitochondria and 50.7% for the chloroplasts). This > 50% GC content is unusual as most mitochondria and plastid genomes are enriched in adenine and thymine. In fact, C-169 is one of only a few eukaryotes to have this property [14].

### Non-random distribution of Zepp retrotransposon

Repeated sequences represent 7.2% (3.5 Mb) of the C-169 genome, a fraction comparable to other sequenced green algae, except for the chlorophyceaen species that have higher repeat contents (Table S3 in Additional file 2). Forty-one percent of the C-169 repeated sequences resemble known repeat families. The most prominent are non-long-terminal-repeat retrotransposons, including Zepp LINEs (16.2%) and retrotransposable elements RTE (5.8%), and SINEs (8.8%) (Table S5 in Additional file 2).

Clusters of nested Zepp retrotransposons were previously found at the termini of C-169 chromosomes [9]. In this present study, we found 26 Zepp clusters in the genome assembly with sizes ranging from 1.5 to 42.3 kb and comprising one to several copies of nested Zepp elements. The 12 complete chromosome scaffolds plus the 4 chromosomes reconstructed by Southern hybridization contain one Zepp cluster each. These clusters most often lie inside chromosomes, where they are relatively distant from telomeres; only two chromosomes have a Zepp cluster in a sub-telomeric position (scaffolds 12 and 23; Figure S2 in Additional file 1). The eight remaining scaffolds corresponding to incomplete chromosomes have either one or no Zepp cluster: two have no Zepp cluster, two have an internally located Zepp cluster and four have Zepp retrotransposons at one end. The distribution pattern of Zepp retrotransposons in the assembled genome assembly suggests that each C-169 chromosome contains strictly one Zepp cluster. Because the average GC content of individual Zepp elements is relatively high (61% GC) compared to the rest of the genome (53% GC), Zepp clusters produce local peaks of GC content within chromosomes.

No sequence in the EST dataset originates from a Zepp element, indicating that they are expressed at very low levels or totally inactivated in the conditions for EST production. In a previous study, Zepp expression was only detected under specific conditions, such as irradiation with an electron beam or following a heat shock [9]. A neutral explanation of the non-random distribution of Zepp retrotransposons is that they integrated into hotspots present as a single copy in each chromosome, for example, centromeric regions. Alternatively, a single Zepp cluster may be indispensable for normal chromosome function.

The report that Zepp elements were constantly present in neoformed minichromosomes supports this hypothesis [10]. These observations suggest a role for Zepp elements or sequences therein in centromeric functions. No tandem satellite repeats, as occurs in the centromeres of many eukaryotes [15], were identified within or in the vicinity of the Zepp clusters. The Zepp elements may be involved in centromere formation in a process similar to the LINE-1 retrotransposons in human neocentromeric regions [16]. The canonical Zepp element possesses two open reading frames encoding reverse transcriptase and Gag-like proteins [9]. BLASTP searches in public databases did not identify significant matches for the Zepp Gag-like protein, while the closest homolog to the reverse transcriptase protein was found in the fungus *Ustilago maydis*. No such Zepp clusters are found in the other green algae genome sequences.

### Conserved synteny with poor gene colinearity

**Figure 2:**
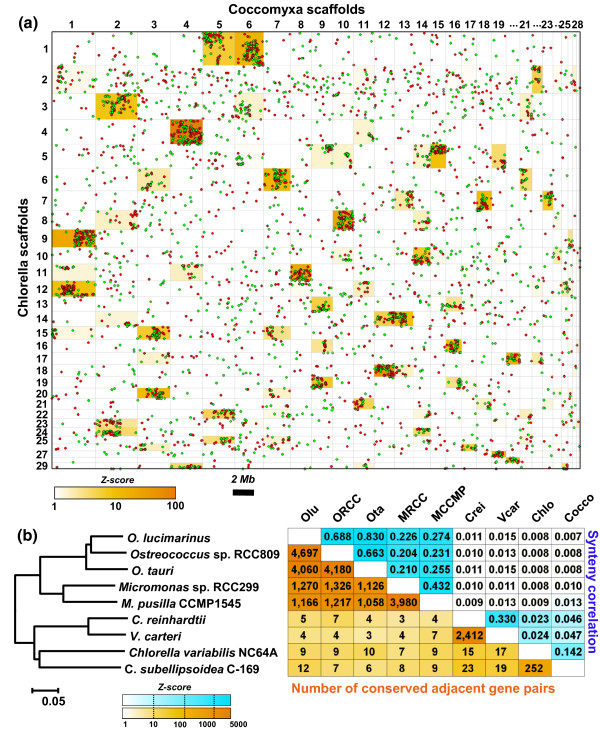
**Levels of conserved synteny between green algae**. **(a) **Dot-plot of 5,232 putative orthologous genes in the genome assemblies of C-169 and *C. variabilis*. Red and green dots show orthologous genes on the same and opposite strands, respectively. The width and length of each box are proportional to the lengths (bp) of the scaffolds determining the box. Scaffolds are organized in decreasing size order. The background color of boxes reflects the statistical significance (Z-score) of the number of orthologous genes (that is, conservation of synteny) shared between pairs of scaffolds relative to a non-syntenic model. The figure shows only the 29 biggest scaffolds of each species. **(b) **Numbers of conserved adjacent gene pairs and synteny correlation coefficients between pairs of sequenced chlorophytes appearing in the phylogenetic tree shown on the left. The maximum likelihood phylogenetic tree of sequenced chlorophytes was computed with the WAG+G+I model from a concatenated alignment of 1,253 orthologous proteins totaling 263,131 gap-free sites. The upper half of the matrix shows the levels of synteny conservation between pairs of genome assemblies as measured by the synteny correlation coefficient [17]. The lower half shows the numbers of pairs of orthologous genes that are adjacent in two genome assemblies. The background color of boxes reflects the statistical significance (Z-score) of the synteny correlation coefficient (blue) and number of conserved adjacent gene pairs (orange) relative to a non-syntenic model. Olu, *Ostreococcus lucimarinus*; ORCC, *Ostreococcus *sp. RCC809; Ota, *Ostreococcus tauri*; MRCC, *Micromonas *sp. RCC299; MCCMP, *Micromonas pusilla *CCMP1545; Crei, *Chlamydomonas reinhardtii*; Vcar, *Volvox carteri*; Chlo, *Chlorella variabilis *NC64A; Cocco, *Coccomyxa subellipsoidea *C-169.

Dot plot analysis of orthologous genes in the genome assemblies of C-169 and *C. variabilis *revealed a conserved synteny (that is, conservation of gene content between homologous chromosomes or segments), although a substantial number of orthologous genes were shared between non-syntenic scaffold pairs (depicted by white boxes in Figure 2a). Within syntenic blocks (orange boxes in Figure 2a), the gene order was highly rearranged, with the dots forming clouds rather than the diagonals expected when orthologous genes locally remain in the same order. In some cases, non-overlapping sub-regions of the same scaffold are in synteny with different scaffolds in the other species (for instance, both C-169 scaffolds 5 and 6 are in synteny with distinct regions of *C. variabilis *scaffold 1), indicating that chromosome fusion, fission or translocation events have occurred since divergence of the two organisms. However, these inter-chromosomal rearrangements are less common than intra-chromosomal rearrangements, resulting in a conserved synteny with poor gene colinearity. We identified 252 conserved pairs of adjacent orthologs (CPAOs; that is, two adjacent genes in one genome with orthologs in an adjacent position in another genome) out of the 5,232 putative orthologs shared between the two species. This is almost ten times less than the number of CPAOs between *C. reinhardtii *and *Volvox carteri *(n_CPAO _= 2,412) and approximately 20 times less than between *Ostreococcus *species (n_CPAO _= 4,060 to 4,697) and between *Micromonas *species (n_CPAO _= 3,980) (Figure 2b). The conservation of synteny as measured by the synteny correlation [17] is primarily restricted to within taxonomic classes (Trebouxiophyceae, Chlorophyceae and Mamiellophyceae) and negatively correlated with genetic distance (Figure S3 in Additional file 1); only a weak, yet significant synteny is conserved between trebouxiophyceaen and chlorophyceaen species and no significant synteny is detected between Mamiellophytes and other algae (Figure 2b).

### Protein family expansion

Annotated proteins of nine sequenced chlorophyte algae (C-169, *C. variabilis *NC64A, *C. reinhardtii*, *V. carteri*, *Micromonas pusilla *CCMP1545, *Micromonas *sp. RCC299, *Ostreococcus *sp. RCC809, *Ostreococcus lucimarinus *and *Ostreococcus tauri*) were organized into 23,507 families based on shared sequence similarity. Except C-169, all these green algae are temperate and live in fresh water (*C. variabilis, V. carteri*), soil (*C. reinhardtii*) or marine water (*Micromonas *and *Ostreococcus *spp.). Assignment of PFAM domains to proteins identified several protein families that have a significantly higher number of proteins in C-169 than in other chlorophyte algae (Table S6 in Additional file 2). The expansion of some of these protein families might reflect adaptation of the alga to a new habitat with extreme conditions.

#### Lipid metabolism

Four over-represented protein families correspond to important steps in lipid metabolism. They include putative type-I fatty acid (FA) synthases, FA elongases, FA ligases and type 3 lipases. In addition, we identified a family of three FA desaturase proteins not found in other green algae (Figure S4 in Additional file 1). These proteins may be involved in adaptive processes that allowed C-169 to survive in the Antarctic environment. These processes include modification of the FA composition (polyunsaturated and branched FAs) of membrane lipids to maintain membrane fluidity at low temperature [18] and production of antifreeze lipoproteins.

**Figure 3:**
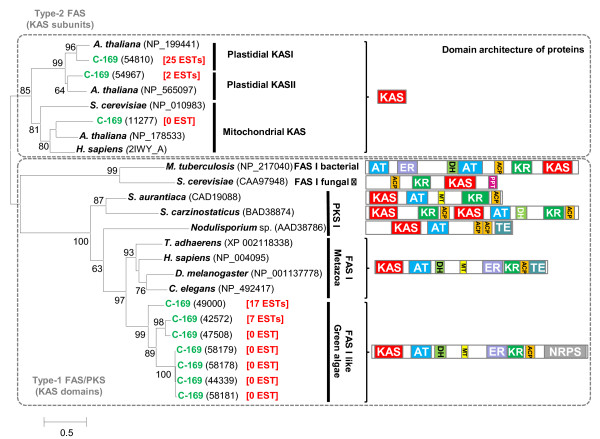
**Maximum likelihood phylogenetic tree of the ketoacyl-ACP synthase (KAS) domains and proteins of fatty acid synthases (FASs) and polyketide synthases (PKSs)**. The phylogenetic tree was constructed using the WAG+G+I substitution model. The multiple-alignment contained 274 gap-free columns. Approximate likelihood ratio test (aLRT) values for branch support are indicated beside branches when aLRT > 50. GenBank accession numbers and protein ids (C-169) are indicated between brackets. For C-169 proteins, the number of ESTs corresponding to the gene is shown in red. The branch length scale bar below the phylogenetic tree indicates the number of substitutions per amino acid site. The functional domain architecture of proteins is shown on the right. Protein domain names are as follows: ACP, acyl carrier protein; AT, acyl transferase; DH, hydroxyacyl-ACP dehydrase; ER, enoyl-ACP reductase; KAS, ketoacyl-ACP synthase; KR, ketoacyl-ACP reductase; MT, methyltransferase; NRPS, non-ribosomal protein synthase terminal domain; PPT, phosphopantetheinyl transferase; TE, thioesterase.

Metazoa synthesize FAs using a large cytoplasmic multidomain FA synthase of type-I (FAS-I) that does not exist in plants. Instead, land plants use a chloroplastic type-II FAS, which is a complex of multiple independent subunits. Surprisingly, C-169 is the sole known Plantae member to encode seven homologs of the metazoan FAS-I. As shown in Figure 3, the nature and organization of FAS-I functional domains are identical in C-169 and Metazoa [19] except for one terminal domain: the thioesterase domain of metazoan FAS-I that releases terminated fatty acid chains is replaced by a domain found at the termini of non-ribosomal peptide synthetases. EST data indicate that at least two FAS-I genes are transcriptionally active at 25°C - the growth temperature at which the EST dataset was generated. Phylogenetic analysis based on the highly conserved ketoacyl synthase (KAS) domains indicates that the C-169 core FAS-I like proteins diverged from their metazoan homologs before the radiation of Metazoa (Figure 3). In contrast, the C-169 non-ribosomal peptide synthetase terminal domain is most closely related to the terminal domains of land plant putative acyl-protein synthetases (Figure S5 in Additional file 1) and has no apparent homologue in Metazoa. C-169 also encodes all subunits of the plastidial type-II FAS (Table S7 in Additional file 2), most of which are tagged by ESTs, suggesting that C-169 synthesizes FA using the plant plastidial pathway. Thus, the core FAS-I system appears to have existed in the common ancestor of plants and Metazoa. In plants, however, the FAS-I system was subsequently lost in most known lineages. Another scenario involving a horizontal gene transfer from an unknown organism is also possible. Although the FAS-I coding sequence is relatively large (10 kb), laterally transferred DNA stretches of larger size have been observed in eukaryotes. In the C-169 lineage, the FAS-I system was retained and associated with a different terminal domain that might allow the system to produce a greater diversity of lipid, polyketide or lipoprotein products. The wider expansion of the FAS-I protein family compared to metazoans suggests that these enzymes played an important role in the adaptation of the alga to its environment.

#### Transporters

Although C-169 can grow on inorganic media, it encodes a large variety of amino acid transporters and amino acid permeases (Table S6 in Additional file 2) that presumably allow the alga to import amino acids from organic extracellular environments such as decomposing algal peat. C-169 also encodes six proteins with high sequence similarity to the plant aluminum-activated malate transporters (ALMT). In land plants, ALMTs mediate tolerance to external toxic aluminum cations by exuding malate that chelates and immobilizes Al^3+ ^at the root surface, thus preventing it from entering root cells [20]. Experimental studies are required to confirm that the algal ALMTs play a similar role in C-169.

#### Cellulose metabolism

Five expanded protein families are putatively involved in polysaccharide and cell wall metabolism (Table S6 in Additional file 2). The production of exopolysaccharides and antifreeze glycoproteins plays an important role in cryoprotection of cold-adapted microorganisms [21]. C-169 encodes 22 putative glycosyl hydrolase proteins belonging to the cellulase family and 9 proteins that match the PFAM glycosyl hydrolase type-9 motif. In this last family, four of the proteins have their glycosyl hydrolase domain attached to a cellulose synthase-like domain that is highly similar to the cellulose synthase of tunicates [22]. In algae, these cellulose synthase-like domains are only found in C-169, *C. variabilis *and *Emiliania huxleyi *and are not orthologous to the cellulose synthases and hemicellulose synthases of land plants (Figure S6 in Additional file 1). Interestingly, the tunicate cellulose synthase gene is also a fusion of a cellulose synthase domain and a glycosyl hydrolase domain (different from the algal glycosyl hydrolase type-9 domain) that has cellulase activity. Based on the identification of both cellulose synthase domains and cellulase domains, we predict that cellulose is a constituent of C-169 cell walls. Additional support for this prediction is that C-169 forms protoplasts after treatment with cellulases and Calcofluor white stains its cell wall [23].

#### Dehydrogenases

C-169 encodes significantly more proteins containing short-chain dehydrogenase/reductase family signatures (PFAM adh_short motif) than other algae (Table S6 in Additional file 2). This large protein family uses a variety of substrates ranging from alcohols, sugars, steroids and aromatic compounds to xenobiotics [24], which is reflected in the wide phylogenetic diversity of short-chain dehydrogenases. Analysis of shared similarity between protein sequences indicates that the higher number of short-chain dehydrogenases in C-169 is essentially due to the specific expansion of a small number of subfamilies (Figure S7 in Additional file 1). Although no hypothesis can be presently advanced as to the functional role of these subfamilies, their specific expansion suggests that they contributed to C-169 adaptation.

### C-169-specific proteins

Of the 2,305 predicted C-169 gene products with no detectable homolog in sequenced chlorophytes, 293 proteins grouped into 196 protein families with significant matches (BLASTP E-value < 1e-5) to other organisms (Table S8 in Additional file 2). Among these proteins are various enzymes putatively involved in defense and detoxification, transport, protection against solubilized dioxygen (for example, DOPA-dioxygenase), cell wall biosynthesis, and carbohydrate metabolism (Table S8 in Additional file 2). Overall, the majority (135/196, 69%) of these C-169-specific protein families have their closest phylogenetic homologs in Streptophytes and other Eukaryotes, which suggest that most of these genes existed in the common ancestor of chlorophytes and were subsequently lost in the Chlorophyceae, Mamiellophyceae and Chlorellaceae lineages. In contrast, bacteria are the closest phylogenetic counterpart of most of the C-169-specific proteins involved in carbohydrate metabolism and defense and detoxification pathways, which suggests that these important biological functions have been enriched by lateral gene transfer from prokaryotes.

Among the most remarkable C-169-specific proteins, we found a translation elongation factor-1α (protid: 54652) that functionally replaces the elongation factor-like EFL present in all the sequenced chlorophytes but C-169 [25]. C-169 is also the only sequenced chlorophyte to encode a putative phospholipase D (Joint Genome Institute (JGI) ID: 38692), an important enzyme involved in stress responses and development in land plants [26]. Furthermore, we found a chalcone synthase-like protein (protid: 45842) whose homologs in land plants and bacteria are involved in the synthesis of secondary metabolites for antimicrobial defense, pigmentation, UV photoprotection, and so on [27].

C-169 encodes a putative RNA-dependent RNA polymerase (RdRP) that resembles *Arabidopsis *homologs required for synthesizing small interfering RNAs (siRNA) involved in RNA silencing [28]. Presumably functioning in the same pathway, C-169 also contains two argonaute-like proteins (AGLs; protid: 56022 and 56024) whose plant homologs bind siRNAs that regulate expression of their target genes. However, homologs to land plant Dicer ribonucleases and dsRNA binding proteins (DRBs), two key components of plant RNA silencing pathways, were absent in C-169. The apparent lack of a complete set of proteins required for RNA silencing suggests that this pathway is either non-functional or extensively modified compared to land plants.

### Proteins involved in CO_2 _concentration

The CO_2_-concentrating mechanism (CCM) allows algae to accumulate internal concentrations of inorganic carbon (Ci; CO_2 _and HCO_3-_) well above the external concentrations in their aqueous environments, thereby promoting efficient photosynthesis and cell growth. Although most cyanobacteria and eukaryotic algae contain a functional CCM, its occurrence in C-169 was in question because another *Coccomyxa *strain symbiotic with a lichen lacks a CCM [29]. However, annotation of the C-169 genome sequence identified 13 orthologs to genes known to be associated with the CCM in *C. reinhardtii *(Table S9 and Supplemental Results in Additional file 2, and Figure S8 in Additional file 1), the most thoroughly studied eukaryotic CCM. These genes include the well characterized CCM-associated genes (for example, *CAH1*, *LCIB*) as well as the master regulator of the *C. reinhardtii *CCM, CIA5/CCM1. These observations suggest that C-169 has a functional CCM.

### Ubiquitous algal genes missing in C-169

Twenty-nine protein families whose genes were found in all sequenced chlorophytes are missing from the C-169 genome assembly (Table S10 in Additional file 2). C-169 does not encode any of the subunits of the glycosyl phosphatidyl inositol (Gpi) transamidase complex (Gpi8p, Gaa1p, Gpi16p, Gpi17p, and Cdc91p), which attach cell surface proteins to the cell membrane via preformed Gpi anchors [30]. Homologs of Gpi8p, Gaa1p, and Gpi16p exist in all other sequenced chlorophytes, while Cdc91p was absent in both C-169 and *C. variabilis*; Gpi17p has not been identified in any algae. C-169 also lacks the Gpi-anchored wall transfer protein (Gwt) that is involved in Gpi-anchor biosynthesis. Thus, the Gpi anchoring system is lacking in this alga.

C-169 lacks a gene encoding a pyruvate phosphate dikinase (PPDK), an enzyme that ensures the interconversion of phosphoenolpyruvate and pyruvate. This protein is ubiquitous among other sequenced chlorophytes and streptophytes. PPDK plays a key role in gluconeogenesis and photosynthesis in C4 plants and is an ancillary glycolytic enzyme in C3 plants [31]. In C-169, phosphoenolpyruvate/pyruvate conversion is apparently performed by three pyruvate kinases (PKs; protein ids: 32937, 61449 and 67234); however, the yield of glycolytically derived ATP per glucose is two in pyruvate kinase-dependent glycolysis and five in PPDK-dependent glycolysis. Thus, C-169 is potentially less effective in producing ATP from glycolysis than other chlorophytes.

Also missing in C-169 are genes encoding dolichyldiphosphatase, mannosyltransferase and carbohydrate kinase, three enzymes involved in glycan metabolism and cell wall maintenance, as well as genes of five families of transporter proteins, including the sodium/sulfate co-transporter, voltage-gated ion channel and maltose exporter families. C-169 lacks a cobalamin-dependent methionine synthase gene but has a cobalamin-independent methionine synthase gene, thus maintaining a functional methionine biosynthetic pathway [32].

C-169 lacks the photosystem 1 (PSI) reaction center subunit N (PsaN) involved in the docking of plastocyanin. Although PsaN is ubiquitous among green plants, it is not essential for phototrophic growth: *Arabidopsis *plants lacking PsaN can assemble a functional PSI complex but show a decrease in the rate of electron transfer from plastocyanin to PSI [33]. Low temperatures induce an excess of electrons going through PSI that are eventually transported to oxygen, thereby generating reactive oxygen species (ROS), which are harmful to the cell [18]. Thus, the unique loss of the PsaN gene in C-169 may be advantageous under cold climates because it may lead to reduced ROS formation.

## Conclusions

The mechanisms of adaptation of life to the extreme environmental conditions encountered in polar regions have interested scientists for a long time. To date, more than 30 psychrophylic microbial genomes have been fully sequenced [34]; C-169 is the first polar eukaryote to have its genome sequenced. Psychrophilic prokaryotes use various adaptive strategies for survival in cold environments, including cold-induced desaturation of fatty acids in membrane lipids, protective mechanisms against increased amounts of solubilized oxygen and ROS, synthesis of antifreeze lipoproteins and glycoproteins, and global change in amino acid composition of encoded proteins to decrease protein structural rigidity [34]. Annotation of the C-169 genome suggests similar adaptive routes (Table 2).

**Table 2:** Adaptive strategies of psychrophilic prokaryotes to cope with low temperatures and potential adaptation in *C. subellipsoidea *C-169

Adaptive strategy	Prokaryotic genes or events involved in the process	C-169-specific genes potentially involved in the process
Increased fluidity of cellular membranes at low temperature	Unsaturated fatty acid (FA) synthesis genes, FA desaturases	Lipid biosynthesis genes, including FA synthase type I, FA desaturases, lipases
Reduction of freezing point of cytoplasm and stabilization of macromolecules	Genes for synthesis of compatible solutes, membrane transporters, antifreeze proteins and ice-binding proteins	Production of antifreeze lipoproteins, exopolysaccharides and glycoproteins: lipid biosynthesis genes, including FA synthase type I and FA ligases; carbohydrate metabolism genes, including glycosyl hydrolases and glycosyl transferases
Protection against reactive oxygen species	Catalases, peroxidases, superoxide dismutases, oxidoreductases	Dioxygen-dependant FA desaturases, DOPA-dioxygenase, loss of the gene encoding photosystem 1 subunit PsaN
Maintain catalytic efficiency at low temperatures	Global change in amino acid composition of encoded proteins to decrease protein structural rigidity	No apparent change in global amino acid composition relative to mesophilic plants and green algae

The adaptive strategies of psychrophilic prokaryotes to cope with low temperatures are modified from Table 1 in [34].

The fact that C-169 has more enzymes involved in the biosynthesis and modification of lipids than other sequenced chlorophytes suggests that this lineage of green alga has adapted to extreme cold conditions through greater versatility of its lipid metabolism, allowing it to synthesize a greater diversity of cell membrane components. These new enzymes and metabolic properties are of potential interest in developing technologies for converting lipids from microalgae into diesel fuel or valuable fatty acids [35]. C-169 encodes specific dioxygenase (DOPA-dioxygenase) and FA desaturases that use dioxygen as a substrate, which, together with the loss of the PsaN gene, can contribute to providing a higher level of protection of the metabolism against ROS. In contrast to psychrophilic organisms that live in permanent cold environments [36], the C-169 proteome exhibits no evidence of systematic bias in amino acid composition relative to the proteomes of other sequenced Plantae that are mesophilic (Figure S9 in Additional file 1). This probably reflects the fact that C-169 lives in Antarctic soils, which withstand wide fluctuations in temperature (typically from -50°C to +25°C). Although C-169 inhabits polar ecological niches and can survive extremely low temperatures, its optimal growth temperature is close to 20°C. Thus, both optimal growth temperature and global amino acid composition indicate that C-169 is not fully specialized to grow in a permanent cold environment.

## Materials and methods

### Organism

C-169 was obtained from the Microbial Culture Collection, National Institute for Environmental Studies, Japan under strain #NIES 2166 *Coccomyxa *sp.

### Genome sequencing and assembly

The C-169 genome was sequenced using the whole genome sequencing strategy. The data were assembled using release 2.10.11 of Jazz, a WGS assembler developed at the JGI. After excluding redundant and short scaffolds from the initial assembly, there was 48.8 Mb of ungapped scaffold sequence. The filtered assembly contained 29 scaffolds, with sizes ranging from 0.112 to 4.035 Mb. The sequence depth derived from the assembly was 12.0 ± 0.15. Pulse field gel electrophoresis studies for assignment of scaffolds to chromosomes were carried out according to Agarkova *et al. *[37]. In addition 28,322 validated ESTs were generated from C-169 cells grown to log phase at 25°C in modified bold basal medium (MBBM). A detailed description of methods is provided in Supplemental Methods in Additional file 2.

### Genome annotation and sequence analysis

The genome assembly of C-169 was annotated using the JGI annotation pipeline, which combines several gene predictors: 1) putative full length genes derived from 7,984 cluster consensus sequences of clustered and assembled C-169 ESTs were mapped to genomic sequence; 2) homology-based gene models were predicted using FGENESH+ [38] and Genewise [39] seeded by BLASTx alignments against sequences from NCBI non-redundant protein set; 3) the *ab initio *gene predictor FGENESH was trained on the set of putative full-length genes and reliable homology-based models. Genewise models were completed using scaffold data to find start and stop codons. Additional gene models were predicted using *ab initio *GeneMark-ES [40] and combined with the rest of the predictions. ESTs and EST clusters were used to extend, verify, and complete the predicted gene models. Because multiple gene models per locus were often generated, a single representative gene model for each locus was chosen based on homology and EST support and used for further analysis. This led to a filtered set of 9,851 gene models with their characteristics supported by different lines of evidence summarized in Tables S1 and S2 in Additional file 2.

All predicted gene models were annotated using InterProScan [41] and hardware-accelerated double-affine Smith-Waterman alignments against SwissProt [42] and other specialized databases like the KEGG (Kyoto Encyclopedia of Genes and Genomes) [43] and PFAM [44]. Finally, KEGG hits were used to map EC numbers [45], and Interpro hits were used to map Gene Ontology terms [46]. In addition, predicted proteins were annotated according to KOG classification [47]. All scaffolds, gene models and clusters, and annotations thereof, may be accessed at the JGI *Coccomyxa *Portal [48] and can also be found in the EMBL/GenBank data libraries under accession number AGSI00000000.

*De novo *identification of repeated sequences was performed by aligning the genome against itself using the BLASTN program (E-value < 1e-15). Individual repeat elements were organized into families with the RECON program using default settings [49]. RECON constructed 2,976 repetitive sequence families from 11,044 individual repeat elements or fragments. Second, identification of known repetitive sequences was performed by aligning the prototypic sequences contained in Repbase v12.10 [50] using TBLASTX. The results of the two methods were combined.

### Protein families

Annotated proteins of nine sequenced chlorophyte algae (C-169, *C. variabilis *NC64A, *C. reinhardtii*, *V. carteri*, *M. pusilla *CCMP1545, *Micromonas *sp. RCC299, *Ostreococcus *sp. RCC809, *O. lucimarinus *and *O. tauri*) were organized into 23,507 families based on shared sequence similarity (BLASTP, E-value < 1e-5) using the Tribe-MCL program [51] with default parameters except inflation parameter set to 1.4. Of those, 6,326 families contained at least one *Coccomyxa *protein, including 1,851 protein families that were found in all 9 species and represent the core protein family set of chlorophyte plants. There were 2,214 protein families containing 2,305 predicted C-169 gene products with no detectable homolog in the other sequenced chlorophytes. Of these, 196 families contained 293 proteins that had significant matches (BLASTP E-value < 1e-5) to other organisms (Table S6 in Additional file 2). Phylogenetic relationships and potential horizontal gene transfer for these 293 proteins were further assessed using the BLAST-EXPLORER program [52], which combines a BLAST search with a suite of tools that allows interactive, phylogenetic-oriented exploration of the BLAST results.

### Phylogenetic analyses

Most phylogenetic analyses were performed through the phylogeny.fr web platform [53]. The Phylogeny.fr pipeline was set up as follows: homologous sequences were aligned with the MUSCLE program [54]; poorly aligned positions were removed from the multiple-alignment using the GBLOCK program [55]. The cleaned multiple alignment was then passed on to the PHYML program [56] for phylogenetic reconstruction using the maximum likelihood criterion. Selection of the best fitting substitution model was performed using the ModelTest program for nucleotide sequences [57] and ProtTest for amino acid sequences [58]. PhyML was run with the approximate likelihood ratio test (aLRT), a statistical test of branch support [59]. This test is based on an approximation of the standard likelihood ratio test, and is much faster to compute than the usual bootstrap procedure while branch supports are generally highly correlated between the two methods.

### Synteny and colinearity

#### Pairwise scaffold synteny

We identified 5,232 putative orthologous gene pairs between C-169 and *C. variabilis *using the reciprocal best blast hit criterion. In Figure 2a, the statistical significance of the number of orthologous genes shared between pairs of scaffolds was estimated by comparison with a non-syntenic model using Z-score statistics. This non-syntenic model was constructed from 1,000 randomized datasets in which the 5,232 orthologous gene pairs were reassociated at random. The number of orthologous genes in each scaffold was kept constant across replicates. For each pair of scaffolds, we calculated the mean and standard deviation of the number of shared orthologous genes in the 100 random replicates. The Z-score was determined by subtracting the mean number of orthologous genes in the non-syntenic model from the observed number of orthologous genes in the real dataset and then dividing the difference by the standard deviation. A Z-score > 3 indicates that the observed number of orthologous genes is significantly higher than in the non-syntenic model with a *P*-value < 0.01.

#### Synteny correlation

In Figure 2b, the measure of synteny correlation established by Housworth and Postlethwait [17] is given by:

$$\rho =\overset{r}{\underset{i=1}{\sum }}\overset{c}{\underset{j=1}{\sum }}\frac{{\left(n_{i,j}-e_{i,j}\right)}^{2}}{n\text{ min}\left\{r-1,c-1\right\}e_{i,j}}$$

where *r *and *c *are the numbers of scaffolds in species A and B, respectively; *n_i,j _*is the observed number of genes on species A scaffold *i *with an ortholog on species B scaffold *j*; *e_i,j _*is the expected number of orthologs shared between species A scaffold *i *and species B scaffold *j *assuming that the genes are scattered independently in the two genomes. That is:

$$e_{i,j}=\left(n_{.,j}\;n_{i,.}\right)/n$$

where *n_i,. _*is the row total of the number of genes on species A scaffold *i *with an ortholog anywhere in species B's genome, *n_.,j _*is the column total of the number of genes on species B scaffold *j *with an ortholog anywhere in A's genome and *n *is the total number of orthologous genes mapped between the two species.

For each pair of genomes, the mean and standard deviation of the synteny correlation in a non-syntenic model was calculated from 1,000 randomized datasets in which the orthologous gene pairs were re-associated at random. These parameters were used to assess the significance of the synteny correlation observed in the real data by means of the Z-score statistics.

### Conserved adjacent gene pairs

For each pair of genomes, the non-syntenic model was constructed by reshuffling the order of all genes (that is, orthologs and non-orthologs) in one of the two genomes, keeping the number of genes in each scaffold constant across replicates. We used 1,000 randomized datasets to estimate the mean and standard deviation of the number of conserved adjacent gene pairs in the non-syntenic model. Z-score statistics was used to assess the significance of the observed number of conserved adjacent orthologous gene pairs in the read dataset relative to the number expected by chance in the non-syntenic model.

## Abbreviations

ALMT: aluminum-activated malate transporter; bp: base pair; CCM: CO_2_-concentrating mechanism; CPAO: conserved pairs of adjacent orthologs; EST: expressed sequenced tag; FA: fatty acid; FAS: fatty acid synthase; Gpi: glycosyl phosphatidyl inositol; JGI: Joint Genome Institute; LINE: long interspersed nucleotide elements; PPDK: pyruvate phosphate dikinase; PsaN: photosystem 1 reaction center subunit N; PSI: photosystem 1; ROS: reactive oxygen species; SINE: short interspersed nucleotide elements; siRNA: small interfering RNA.

## Competing interests

The authors declare that they have no competing interests.

## Authors' contributions

GB, IA, TP, JG, AK, AB, JMC, and JVE wrote the article. GB, IA, TP, JG, AK, AB, IL, EL, SL, JP, AS, AL, MB, DD and JS performed research and analyzed data. GB, DW, TY, JMC, IG, and JVE designed research. All authors have read and approved the manuscript for publication.

## Supplementary Material

Additional file 1 **Supplemental figures**. This PDF document contains supplementary Figures S1 to S9. Click here for file

Additional file 2 **Supplemental data and tables**. This PDF document contains Supplemental Methods, Supplemental Results, Supplemental References, Supplemental Tables S1 to S10 and legends of Supplemental Figures S1 to S9. Click here for file
