# Supplementary material for: The genome of the polar eukaryotic microalga Coccomyxa subellipsoidea reveals traits of cold adaptation
Source: Genome Biol. 2012 May 25;13(5):R39. doi: 10.1186/gb-2012-13-5-r39 (PMC3446292; doi:10.1186/gb-2012-13-5-r39)
Supplement: Additional file 1 — Supplemental figures. This PDF document contains supplementary Figures S1 to S9. [file gb-2012-13-5-r39-S1.PDF]

Supplemental Figure 1

A

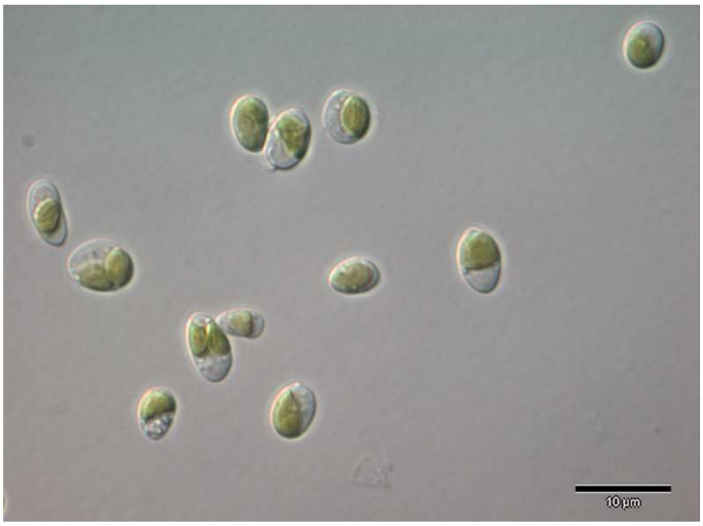

1

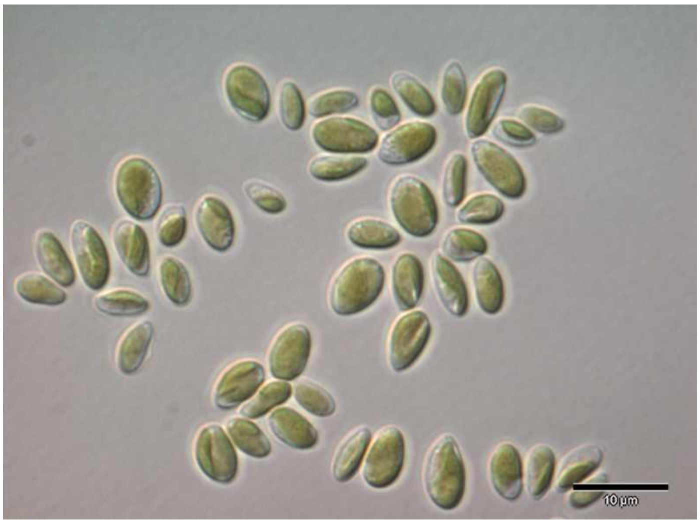

2

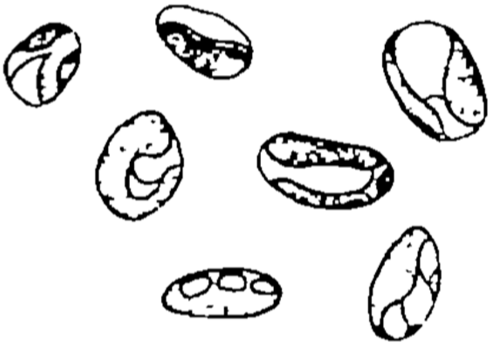

3

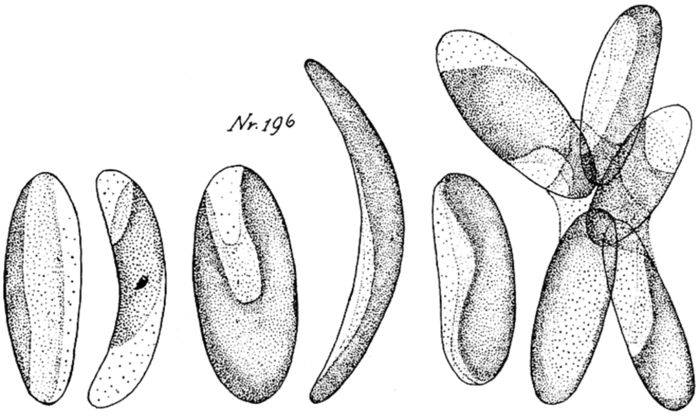

4

Supplemental Figure 1 (continued)

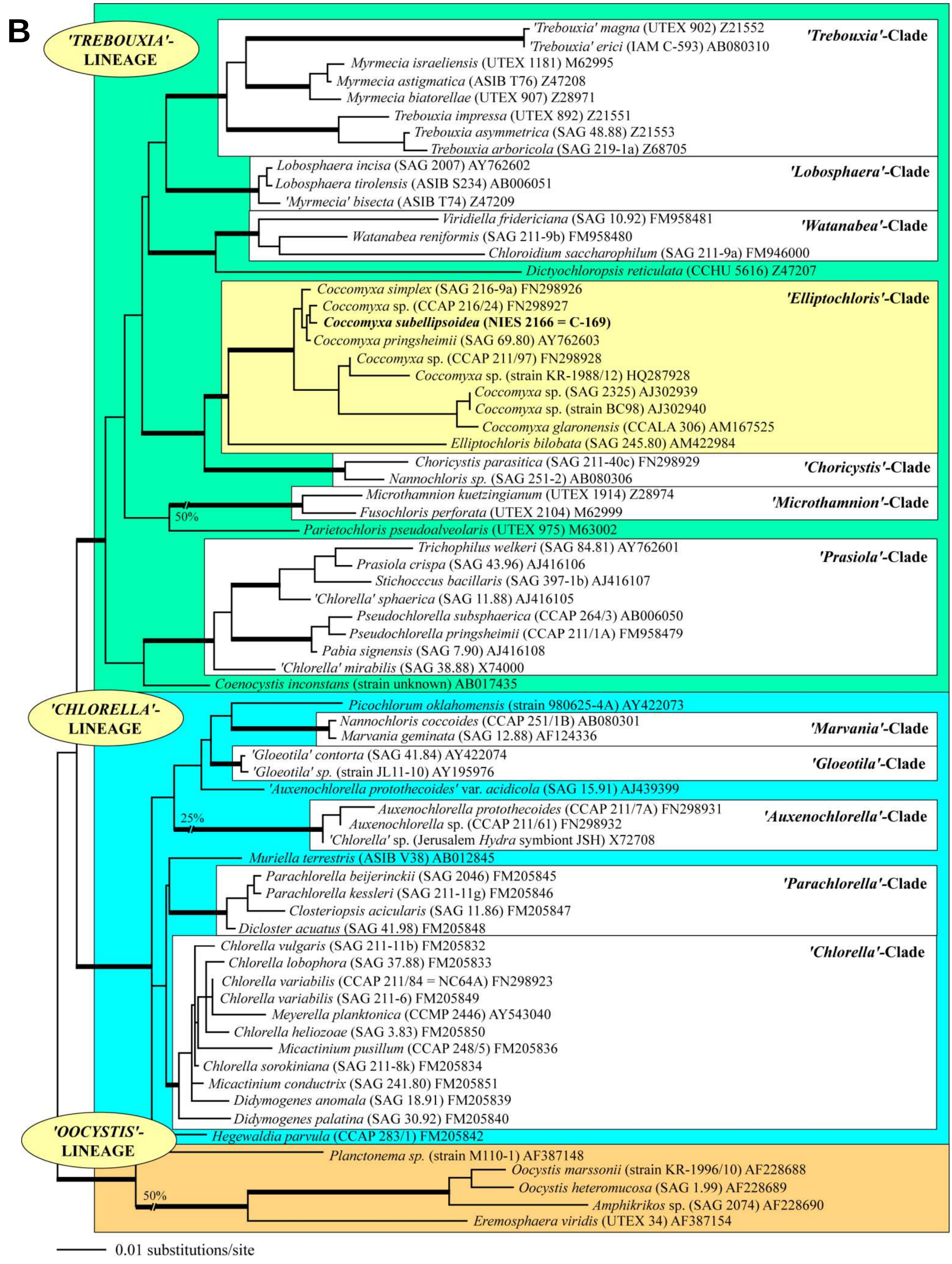

Supplemental Figure 1 (continued)

C

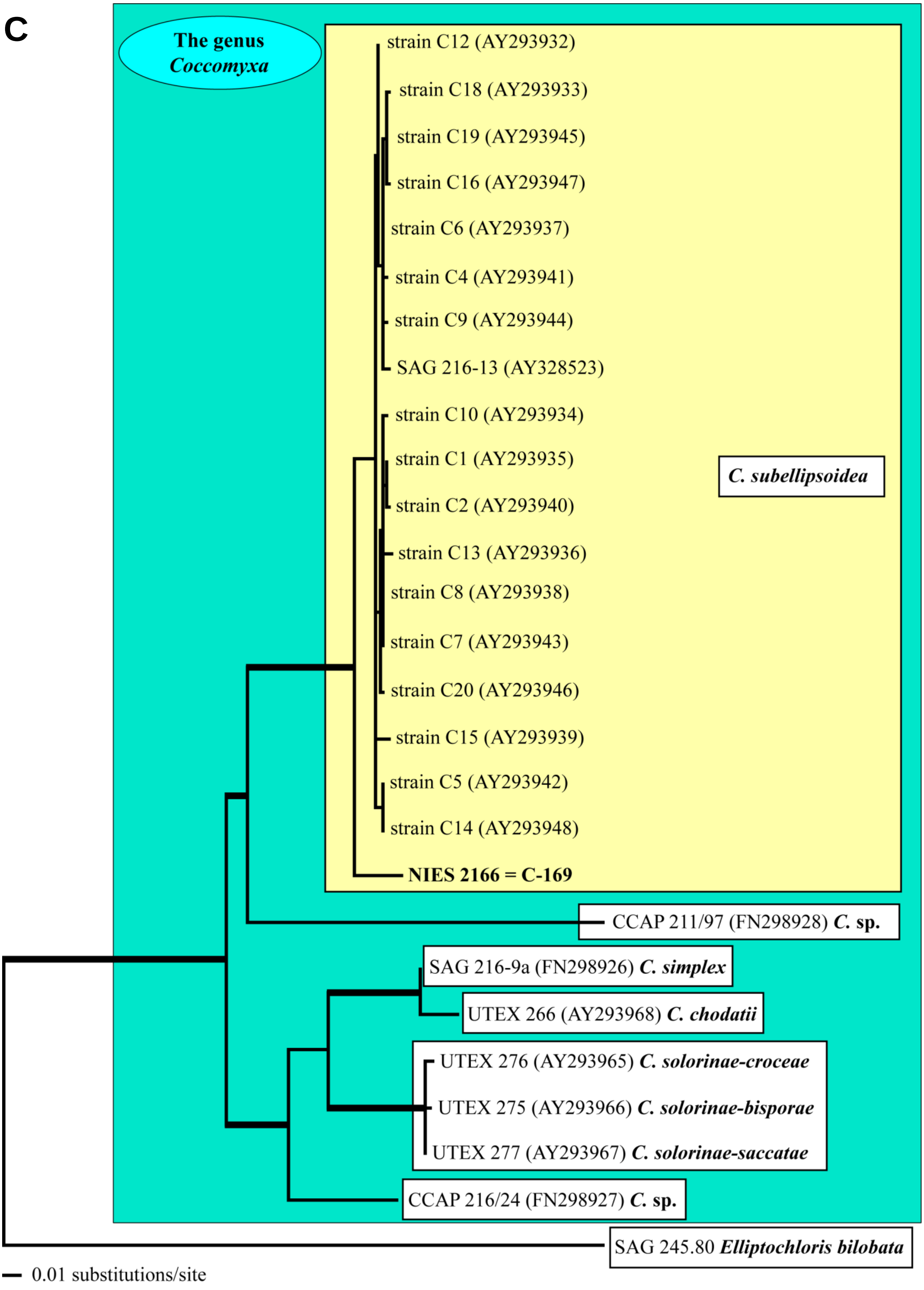

Supplemental Figure 1 (continued)

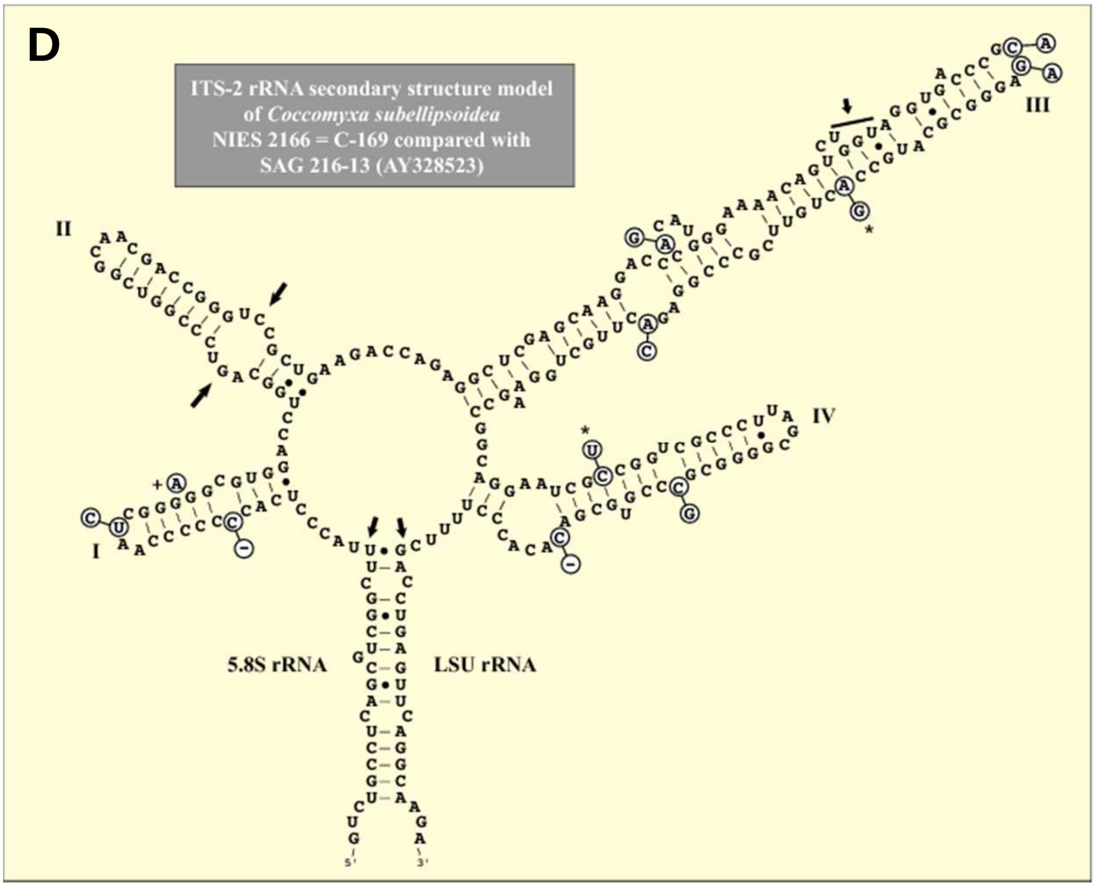

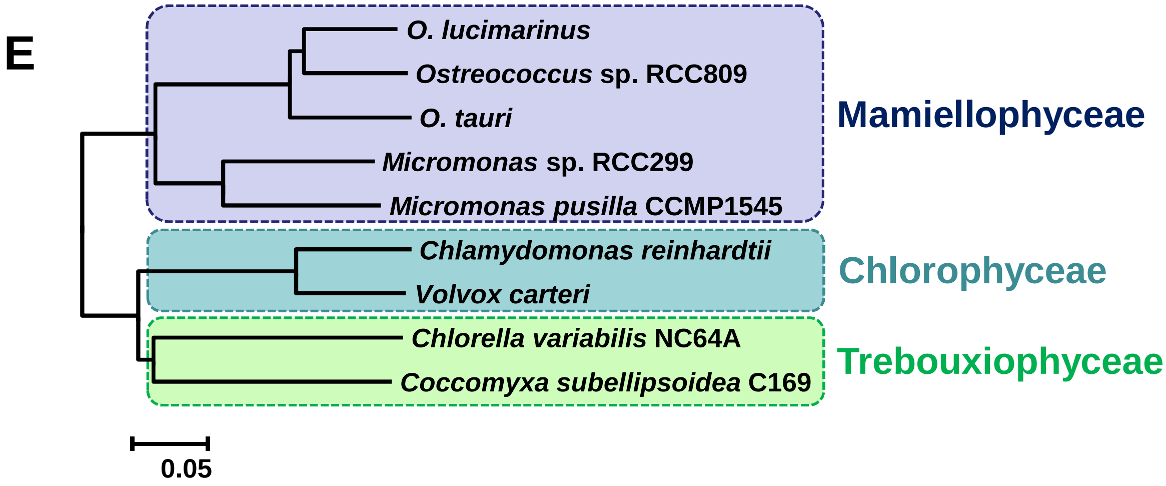

Supplemental Figure 1 (continued)

Supplemental Figure 1: A taxonomy study of C-169

A: Morphology of C-169 (1) compared to the authentic strain of *Coccomyxa subellipsoidea* SAG 216-13 (2), the original figures published by Acton (3) and Jaag (4) reporting the first formal descriptions of *C. subellipsoidea*.

B: Molecular phylogeny of the Trebouxiophyceae based on SSU rDNA sequence comparisons. The phylogenetic tree shown was reconstructed using the maximum likelihood method based on a data set of 1746 aligned positions of 70 taxa. Bootstrap values (> 70%) of the neighbour-joining (using the GTR+I+G model, 1000 replicates), and maximum parsimony (1000 replicates) were marked with branches in bold in the tree. Strain and accession numbers are given after the species name. The clade designation follows Pröschold et al. (27). The *Oocystis*-clade was used as out-group.

C: Molecular phylogeny of the *Elliptochloris*-clade based on ITS-1, 5.8S, and ITS-2 rDNA sequence comparisons. The phylogenetic tree shown was inferred by maximum likelihood method based on a data set of 784 aligned positions of 27 taxa. Bootstrap values (> 70%) of the neighbour-joining (using the GTR+G model, 1000 replicates), and maximum parsimony (1000 replicates) were marked with branches in bold in the tree. Strain and accession numbers are given after the species name. *Elliptochloris bilobata* was used as out-group.

D: Comparison of the ITS-2 secondary structure of strains belonging to *Coccomyxa subellipsoidea* Acton emend Jaag. The C-169 ITS-2 sequence is presented with differences with that of SAG 216-13, the authentic strain of *C. subellipsoidea*, highlighted. Single base changes are encircled. The hemi-compensatory base changes are marked with an asterisk. The numbering of the helices followed the designation described by Coleman & Mai (1997). The arrows in Helix II and III mark the RNA processing sites, which are pyrimidine-pyrimidine mismatch in Helix II (usually a UxU mismatch after the fifth or sixth base pairs; in our case a bulge of three unpaired base pairs after the fourth base pair) and the GGU at the 5' site of Helix III (present in all Viridiplantae: Green algae and higher plants). The two arrows at the 5.8S-LSU stem mark the cutting site, where the ITS-2 is cut during the RNA processing.

E: A maximum likelihood phylogenetic tree of sequenced chlorophytes. The phylogenetic tree was computed using the WAG+G+I model of amino acid substitution from a concatenated alignment of 1,253 ubiquitous orthologous proteins totalizing 263,131 gap-free sites. All interior nodes received 100% bootstrap support (aLRT).

Supplemental Figure 2

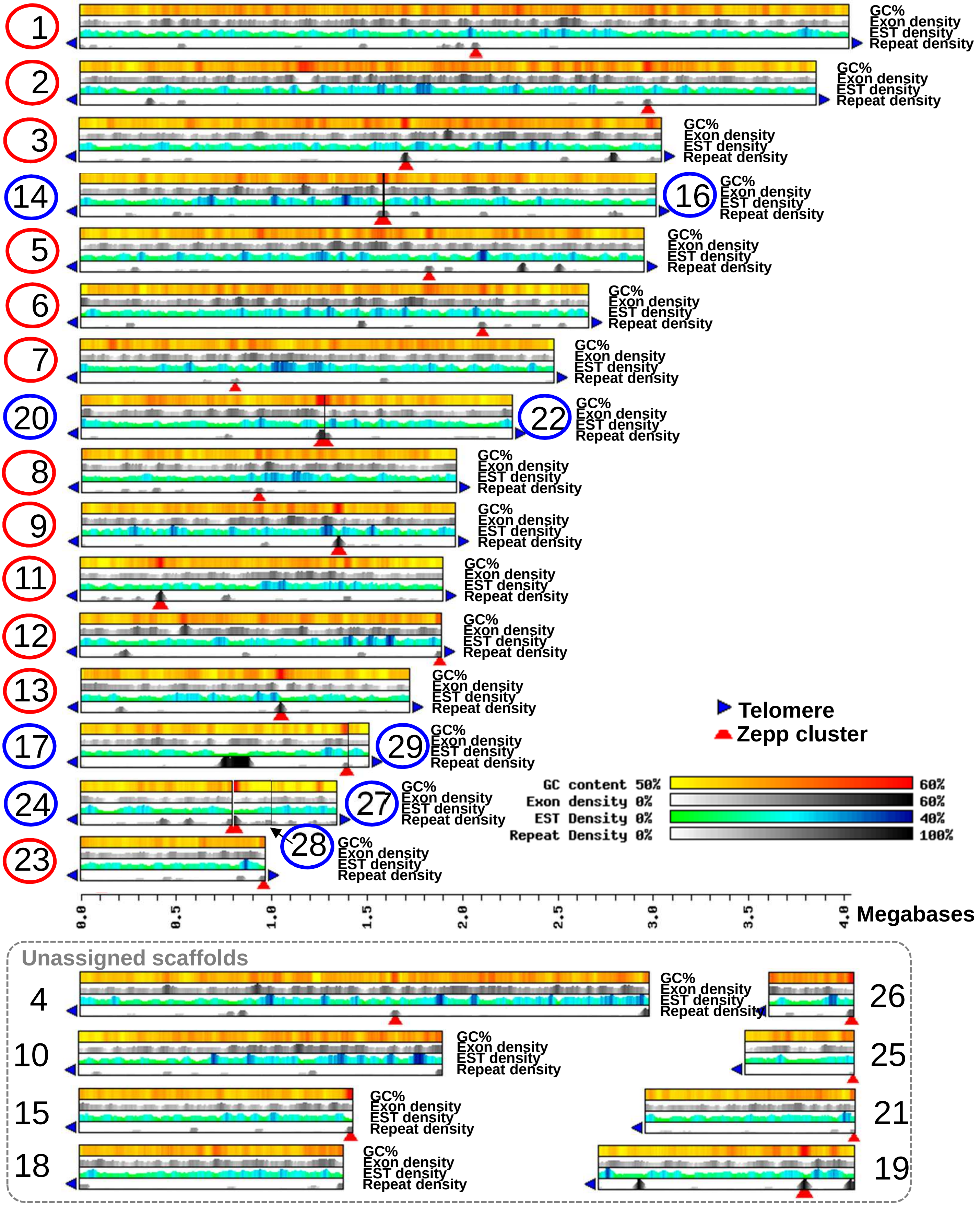

Supplemental Figure 2 (continued)

Supplemental Figure 2. General characteristics of the *C. subellipsoidea* C-169 genome assembly

This figure represents the 29 scaffolds of the C-169 genome assembly. GC percentage, exon density, EST density, and repeat density were calculated in 40-kb sliding windows with a step of 5 kb. Density was calculated as the percentage of nucleotide in the window covered by the relevant feature (i.e., exon, EST, or repeat sequence). Scaffolds with numbers circled in red correspond to chromosomes that were fully reconstructed from sequence assembly. Scaffolds with numbers circled in blue form complete chromosomes joined from hybridization evidence. Blue and red triangles represent telomeric repeat arrays and ZEPP retrotransposon clusters.

Supplemental Figure 3

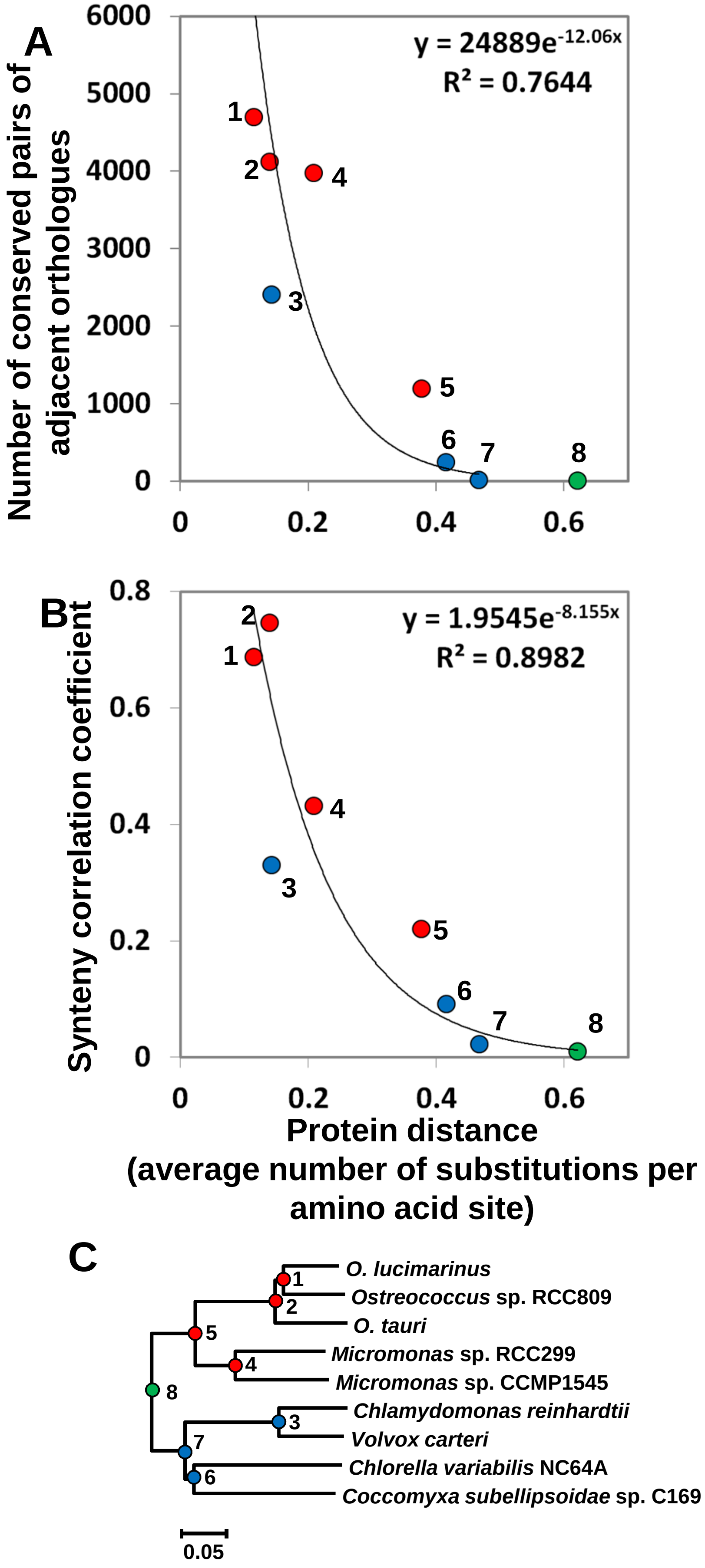

Supplemental Figure 3 (Continued)

Supplemental Figure 3: Synteny correlation and conservation of gene order are inversely correlated with evolutionary distance

A: Protein distance versus number of conserved pairs of adjacent orthologues (CPAO) calculated between pairs of organisms in the chlorophyte phylogenetic tree shown in fig. S4C. For nodes with more than 2 leaves, we plotted the averages of protein distances and numbers of CPAO between all possible pairs of organisms sharing the same last common ancestor. Pairwise protein distances were calculated from a concatenated alignment of 1,253 orthologous proteins shared by all sequenced chlorophytes (totalizing 263,131 gap-free sites). Pairwise distances were calculated using the maximum likelihood method implemented in the PUZZLE program and the WAG+G substitution model. An exponential regression line is shown in black, with equation and correlation coefficient indicated on the top right side of the plot.

B: Same as A with synteny correlation coefficients plotted on the y-axis.

C: Phylogenetic tree of the sequenced chlorophytes with nodes numbered and shaded according to A and B.

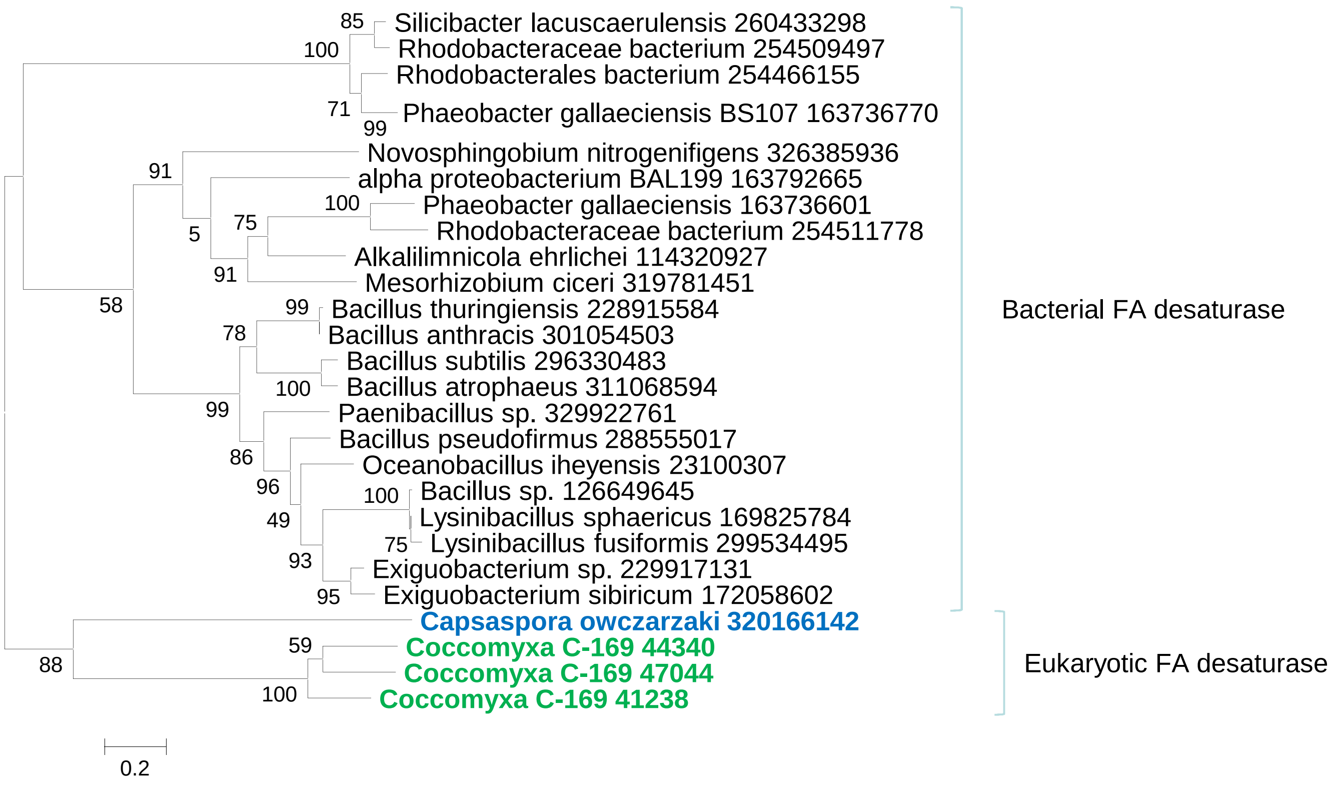

Supplemental Figure 4: Maximum likelihood phylogenetic tree of fatty acid desaturase proteins specific to C-169

The multiple-sequence alignment contained 240 gap-free sites. The phylogenetic tree was reconstructed using the WAG+I+G model of substitutions and the PHYML program. Approximate likelihood ratio test values for nodes are indicated beside branches. Genbank identification numbers are indicated after species names, except for C-169 proteins where JGI protein identification number are given.

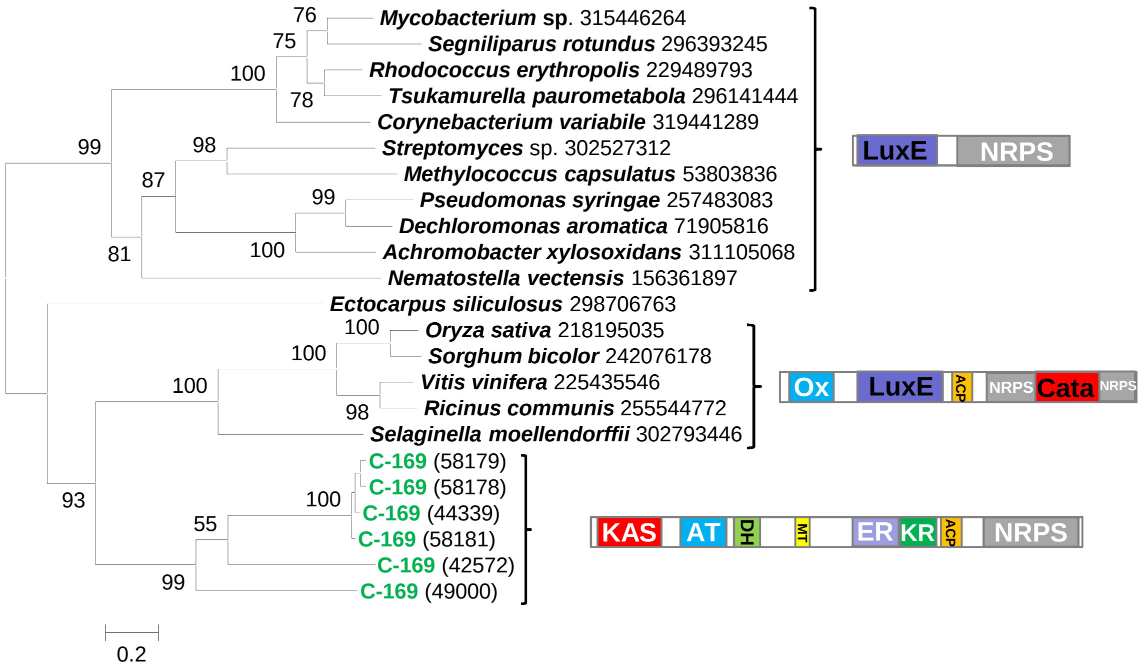

Supplemental Figure 5: Maximum likelihood phylogenetic tree of non-ribosomal protein synthase (NRPS) domains.
The phylogenetic tree was constructed using the WAG+G+I substitution model and the PHYML program. The multiple-sequence alignment contained 494 gap-free columns. Approximate likelihood ratio test (aLRT) values for branch support are indicated beside branches when aLRT >50. Genbank identification (gi) numbers and JGI protein ids (C-169) are indicated between brackets. The functional domain architecture of proteins is shown on the right. Domain names are as follows: ACP: Acyl Carrier protein; KAS: ketoacyl-ACP synthase; AT: acyl transferase; DH: hydroxyacyl-ACP dehydrase; MT: methyltransferase; ER: enoyl-ACP reductase; KR: ketoacyl-ACP reductase; NRPS: non-ribosomal protein synthase terminal domain; LuxE: LuxE family peptide synthase; Ox: oxydoreductase; Cata: catalase.

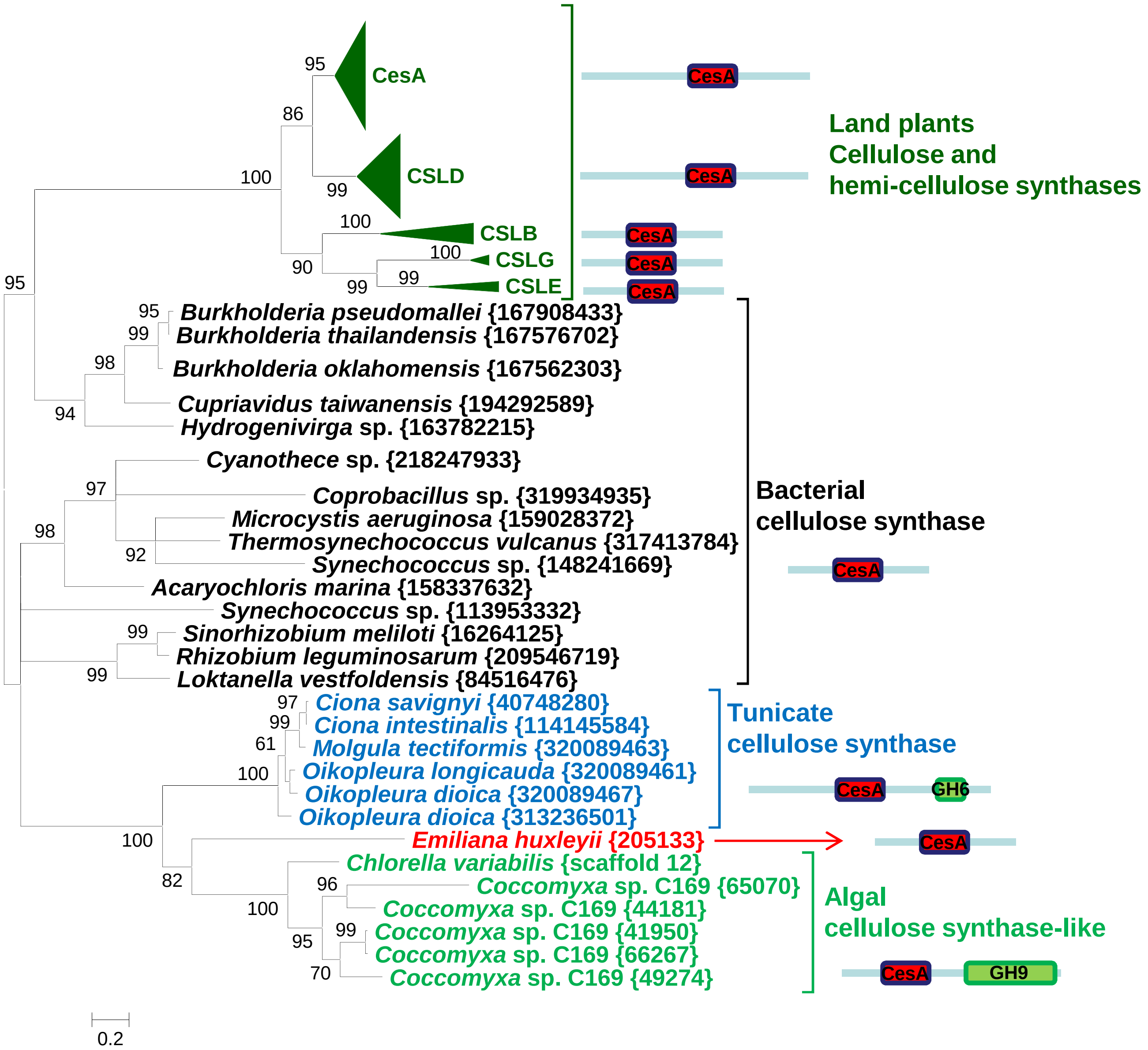

Supplemental Figure 6: Maximum likelihood phylogenetic tree of cellulose synthase-like (CesA) protein domains
The multiple-sequence alignment contained 340 gap-free sites. The phylogenetic tree was reconstructed using the WAG+I+G substitution model and the PHYML program. Approximate likelihood ratio test (aLRT) values >50 are indicated beside branches. Branches with aLRT<50 were collapsed. Genbank and JGI ids of sequences are given between curly brackets, respectively. The *Chlorella variabilis* protein missing from the current version of the genome annotation was identified on the scaffold 12 by a TBLASTN search using the C-169 protein as query. A scheme of the general protein domain architecture is given for each major sequence clade; CesA: Cellulose Synthase-like domain (cd06421), GH6 and GH9: Glycosyl hydrolase type 6 (PF01341) and type 9 (PF00759) respectively. The algal cellulose synthase-like domains featured motifs common to UDP-dependent, polymerizing β-glycosyltransferases in the GT-2 family, wherein the conserved "D, DxD" and "D, QxxRW" residues form the binding sites for the donor UDP and acceptor sugar, respectively (Charnock et al., 2001). In contrast to the other proteins in the tree, the algal proteins did not contain the "KAG" motif of unknown function expected of the cellulose synthase subfamily (Saxena and Brown, 2000; Richmond and Somerville, 2000). In replacement, *C. variabilis* and C-169 have a "KSG" or "KSA" motif.

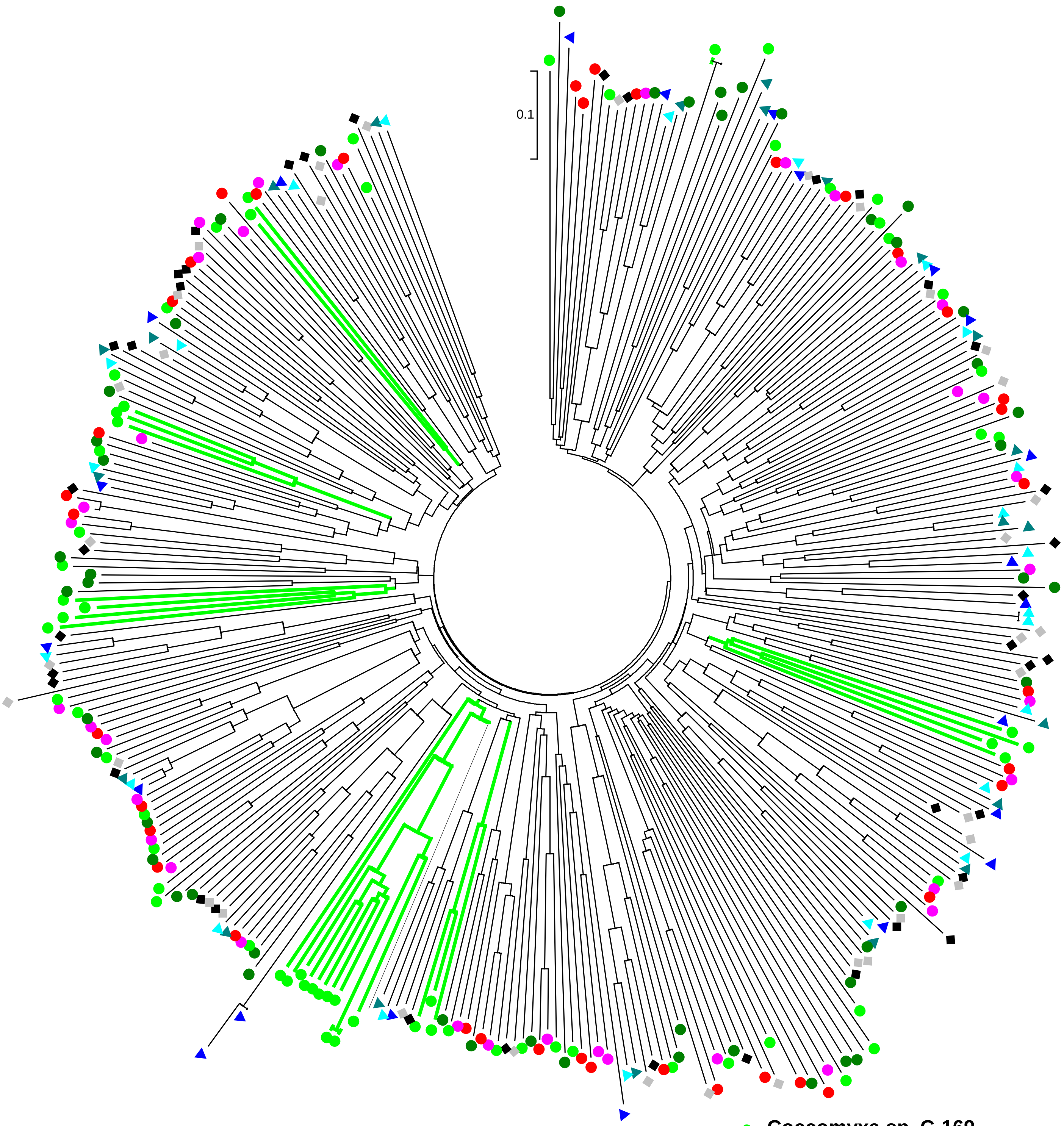

Supplemental Figure 7. Similarity tree of algal short-chain dehydrogenase protein domains

Proteins of sequenced chlorophytes that have significant match to the adh_short PFAM motif were aligned using MUSCLE. A neighbour joining tree was constructed from the multiple-sequence alignment using CLUSTALW and P distance, without removing gapped positions. This similarity tree is an approximation of the real phylogenetic tree. A robust phylogenetic tree could not be reconstructed using a standard procedure due to a high number of gapped positions in the multiple-alignment. Proteins are symbolized by shaded shapes to indicate to what species they belong to. See the color code caption in the figure for more details. Green branches highlight putative subfamilies that are specifically expanded in the C-169 lineage.

```
                                                        ▼
C_reinhardtii     MEALDAQDSLQLDVV--SPSARPAAAGGDKRDPERFYCPYP---------
Ch_NC64A          MPNTGKRA--------EPPTDEEKARIVTGPDPDRFYCPHPGEAMGAPSE
Co_C169           MQQKQ---------------ARTQSGALRERDPDRFYCPYP---------
V_carteri         MELDAPVDSQQLDGIAPSPITQPTSGKDGAKDPERFYCPYP---------
                  *                    .  :      **:*****:*
                           ▼            ▼                      ▼  ▼
C_reinhardtii     --------GCNRSFAELWRLKVHYRAPPDIRGSGKERGHGTELTHCPKCG
Ch_NC64A          GPGAAAAHGCNRSFAELWRLKVHFRAPPDVRGSGKERGHGTELKFCPKCG
Co_C169           --------NCTRSFAELWRLKVHYRAPPDARGSGKERGHGCELQFCPKCA
V_carteri         --------GCNRSFAELWRLKVHFRAPPDIRGSGKERGHGTELTHCPKCG
                          .*.************:***** ********** ** .****.
                          ▼  ▼
C_reinhardtii     KTLKPGKHHVGCSGGKSAPRQTASKRNRTGAD--DADEAVPGSPHSKHVR
Ch_NC64A          KDLRPGKHHVGCSAGKSAPRQAA-KRQRQQQMST-------TTESAQGLT
Co_C169           KELKAGKHHVGCFAGRAGAKQAA-KRSKAQEDFG-------KTQTVEIDM
V_carteri         KTLKPGKHHVGCSGSKTSARQAN-KRTRVAGEAELPDEAPTSSHLAKHVK
                  * *:.******* ..::..:*:  ** :              :   :
```

Supplemental Figure 8: Multiple alignment of *Chlamydomonas reinhardtii* CIA5 with putative orthologs from C-169, *Chlorella variabilis* and *Volvox carteri*.

Only the N-terminal portion of alignments are shown. Residues critical for zinc binding and/or response to carbon-limited conditions in *C. reinhardtii* are marked (▼). The *Volvox carteri* protein sequence was acquired from Fukuzawa et al., 2001 .

Supplemental Figure 9

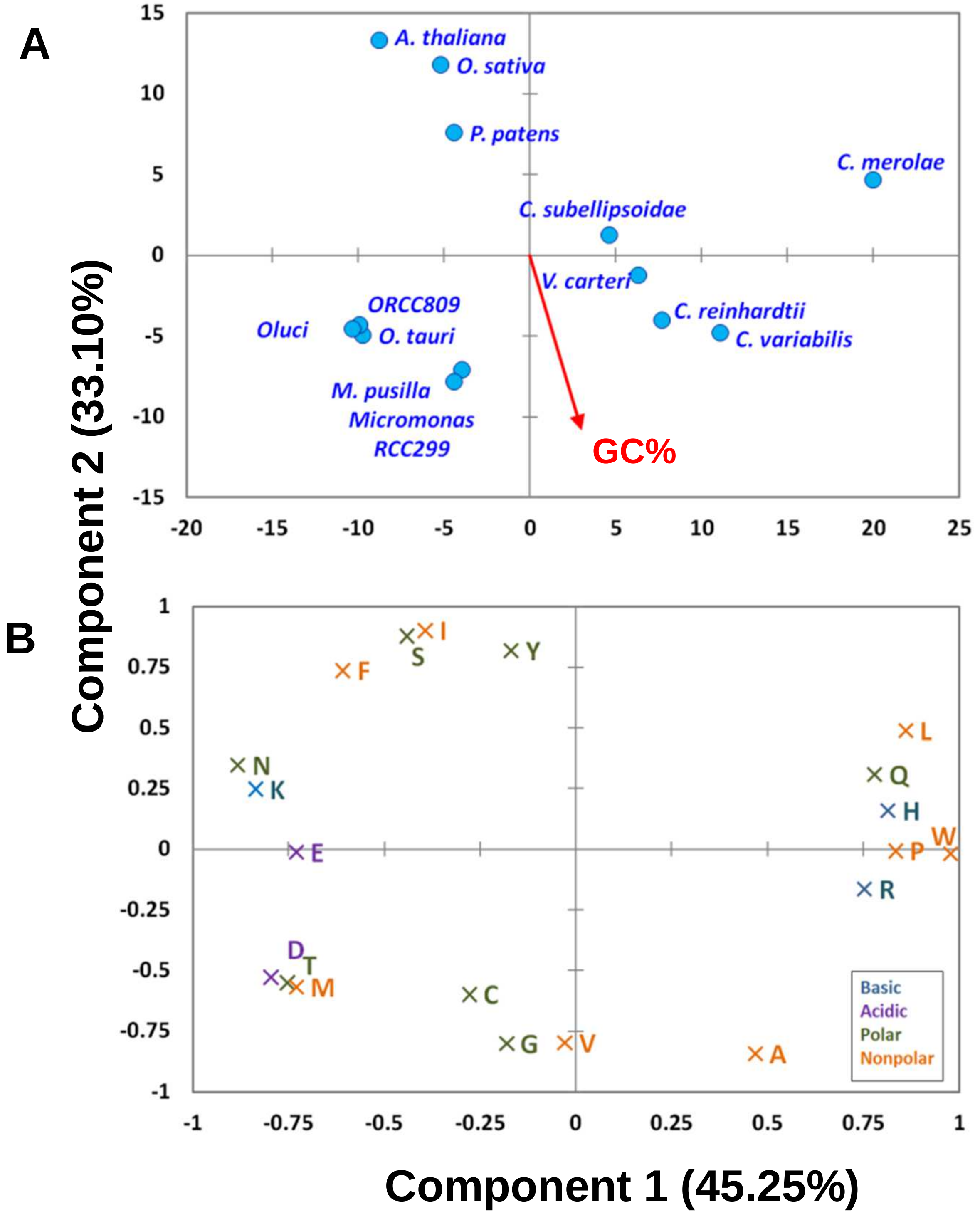

Supplemental Figure 9 (Continued)

Supplemental Figure 9: Principal component analysis (PCA) of amino acid composition in plantae proteomes.

(A) Component scores for organisms; (B) component loadings for amino acids. PCA can be applied to a multivariate data set in order to reduce the complexity of the data and to determine whether there are underlying trends that explain the observed variation. The first two principal components (PCs) accounted for 78.3% of the variance in amino acid composition. PC 2 correlated strongly with genome GC content (correlation coefficients for PC1 and PC2 scores vs. GC content were 0.25 and -0.91, respectively). The influence of GC content on PCs 1 and 2 was apparent, with amino acids encoded by high GC codons (Gly, Ala, Arg, Val, Pro) appearing on the bottom right of the plot while amino acids encoded by low GC codons (Tyr, Lys, Phe, Ile, Asn) appearing on the top left of the plot.
The amino acid composition in proteins from C-169 is not sufficiently different to distinguish it from the mesophilic Plantae species. In fact, the amino acid compositions of Streptophytes (*P. patens, O. sativa, A. thaliana*), Chlorophyceae (*C. reinhardtii, V. carteri*) and Trebouxiophyceae (*C. variabilis, C. subellipsoidea*) appears to be mainly influenced by the genome GC content. In contrast, the presence of the unicellular red alga *Cyanidioschyzon merolae* as an outlier on top right side of the plot can be attributed to the overrepresentation of basic amino acids (Histidine and arginine), which may represent an adaptation to highly acidic environments. Furthermore marine *Ostreococcus* and *Micromonas* species are clustered in the bottom left side of the plot in particular because they have an overrepresentation of acidic amino acids, which is a known adaptation of halophilic species (Kennedy et al., 2001).
