# Supplementary material for: The genome of the polar eukaryotic microalga Coccomyxa subellipsoidea reveals traits of cold adaptation
Source: Genome Biol. 2012 May 25;13(5):R39. doi: 10.1186/gb-2012-13-5-r39 (PMC3446292; doi:10.1186/gb-2012-13-5-r39)
Supplement: Additional file 2 — Supplemental data and tables. This PDF document contains Supplemental Methods, Supplemental Results, Supplemental References, Supplemental Tables S1 to S10 and legends of Supplemental Figures S1 to S9. [file gb-2012-13-5-r39-S2.PDF]

# Additional File 1

## Content

1 Supplemental Material and Methods ........ 1
1.1 *C. subellipsoidea* Genomic DNA Preparation ........ 1
1.2 Pulse Field Gel Electrophoresis and Southern Blot hybridization ........ 2
1.3 Genome sequencing, assembly and finishing ........ 3
1.4 cDNA library construction and sequencing: ........ 3
1.5 EST sequence processing and assembly: ........ 4
1.6 Phylogenetic analyses of rDNA sequences ........ 5
2 Supplemental Results ........ 6
2.1 Morphology, taxonomy and history of strain C-169 ........ 6
2.2 Telomeric repeats and Chromosome count ........ 6
2.3 Carbon Concentrating Mechanism - Conservation Across Algal Species ..... 7
3 Supporting Tables ........ 9
Table S1 ........ 9
Table S2 ........ 9
Table S3 ........ 10
Table S4 ........ 10
Table S5 ........ 11
Table S6 ........ 12
Table S7 ........ 13
Table S8 ........ 14
Table S9 ........ 19
Table 10 ........ 20
4 Supporting Figure Legends ........ 21
5 Supporting References ........ 25

# 1 Supplemental Material and Methods

## 1.1 *C. subellipsoidea* Genomic DNA Preparation

C-169 was streaked onto a modified Bold's Basal medium (MBBM) plate and a single colony was grown to log phase (1 - 2 X $10^7$ cells/ml) in liquid MBBM medium. The cells were harvested by centrifugation for 6 min at 5,000 g 4$^o$C, flash frozen with liquid nitrogen, and stored at -80$^o$C. The cell pellets, containing a total of 3 X $10^{10}$ cells, were then processed using a modification of the standard operating procedure /protocol from JGI for bacterial genomic DNA isolation using CTAB, version number 2.

Cell pellets were thawed in 1X TE buffer, supplemented with 0.5% SDS and 100 µg/ml proteinase K, and incubated at 53$^{o}$C overnight (16 h). NaCl was added to 0.6 M. Pre-warmed CTAB/NaCl solution was added for a final concentration of 28 mM CTAB/0.65 M NaCl and incubated at 65$^{o}$C for 1 h. The sample was extracted with chloroform:isoamyl alcohol (24:1) followed by phenol:chloroform:isoamyl alcohol (25:24:1). Nucleic acids were precipitated with isopropanol, washed with 70% EtOH, and dried. The pellets were resuspended in 1X TE buffer, supplemented with RNAse A to 100 µg/ml and incubated at 37$^{o}$C for 30 min. The total DNA sample was extracted with phenol:chloroform:isoamyl alcohol (25:24:1), precipitated with 0.3 M NaOAc and 2 1/2 vol. EtOH, dried and resuspended in 1X TE buffer.

Total DNA was centrifuged on 40-60% CsCl gradients equilibrated with 1X TE, pH 8.0 buffer containing 1 µg/ml Hoechst 33258 dye to enrich for nuclear DNA. The upper bands containing chloroplast DNA were removed and the lower bands containing nuclear DNA were collected with a wide-mouth pipet tip. The Hoechst dye was extracted from the DNA twice with an equal volume of CsCl/TE-saturated isopropanol. The samples were diluted with 1X TE and the DNA was precipitated with 0.3 M NaOAc and 2 vol. of EtOH at -20oC, washed with 70% EtOH, dried, and resuspended in a total of 800 µl 1X TE, pH 8.0.

The quality of the purified nuclear-enriched genomic C-169 DNA was monitored with a wavelength absorbance scan and by electrophoresis on a 0.8% 1X TBE agarose gel compared to varying amounts of lambda phage DNA.

## 1.2 Pulse Field Gel Electrophoresis and Southern Blot hybridization

The C169 karyotype was determined by using pulse field gel electrophoresis (PFGE). Intact chromosomal DNAs were isolated as described [1,2]. Briefly, 100 ml of actively growing C-169 cells were harvested from 4-day old cultures by centrifuging at 5000 × g for 5 min, washed 3 times with ice cold TE buffer amended with 50 mM EDTA and then re-suspended in 0.5 ml of TE buffer at a concentration of 0.6-1.0 × $10^{9}$ cells/ml. The re-suspended cells were mixed with an equal volume of 2% low melting point agarose (Bio-Rad) in TE buffer at 45°C, poured into plug molds (Bio-Rad, Hercules, CA), and placed at 4°C for 15 min to solidify. Agarose blocks were incubated in approximately 2 ml of 1 mg/ml proteinase K in DB solution (250 mM EDTA, pH 9.5; 1% N-lauroylsarcosine) for 24 h. After digestion, samples were washed two times for 30 min with DB solution and cut into small pieces that fit into gel wells. Samples were sealed with 0.8% low melting point agarose at 45°C in electrophoresis buffer. Algal chromosomes were separated using a CHEF-DR II unit (Bio-Rad, Hercules, CA) in 0.8% agarose in 0.5 TBE buffer. Electrophoresis was typically performed for 96 hrs with pulse time ramping from 1200 to 700 sec. The voltage was 2 V/cm during the first 48 hrs and then it was increased to 3 V/cm. In some runs the length of pulse time voltage, concentration of agarose and electrophoresis time was varied slightly to resolve targeted chromosome. Separated chromosomes were transferred (using the Bio-Rad protocol for PFGE gel transfer) onto nylon membranes (GeneScreen Plus, PerkinElmer, Boston, MA).

The scaffold primers used to make DNA probes were designed to generate unique DNA fragments of 400 bp by PCR amplification. A DNA probe was generated for each of the 17 scaffolds representing chromosome pieces (scaffolds 4, 10, 14, 15, 16, 17, 18, 19, 20, 21, 22, 24, 25, 26, 27, 28 and 29). DNA probes were labeled with [$^{32}$P] dATP using a random primer kit (NEBlot, New England Biolabs, Beverly, MA).

Southern blot analysis was performed according to the membrane manufacturer's manual (PerkinElmer).

Nine scaffolds were assembled into 4 chromosomes based on colocalization of hybridizing bands. Three of the chromosomes were formed by 2 scaffolds (Scaffold_14 and Scaffold_16 [3.0 Mb]; Scaffold_20 and Scaffold_22 [2.3 Mb]; Scaffold_17 and Scaffold_29 [1.5 Mb]). One chromosome contained 3 scaffolds (Scaffold_24, Scaffold_27 and Scaffold_28 [1.4 Mb]). Paired scaffolds exhibited the same repeated sequences at their joined ends, indicating that the failure to assemble entire chromosomes resulted from the difficulty to assemble repeated sequences. The remaining 8 scaffolds could not be resolved into individual chromosomes because they hybridized to overlapping bands near the top of the electrophoresis gel.

## 1.3 Genome sequencing, assembly and finishing

The C-169 genome was sequenced using WGS strategy. Four libraries with insert sizes of 2-3 KB, 6-8 KB, and 35-40 KB were used, which resulted in the following dataset:

364,160 2-3 KB reads, containing 407 MB of sequence.

415,218 6-8 KB reads, containing 332 MB of sequence.

74,496 35-40 KB reads, containing 58 MB of sequence.

The sequenced paired end reads were screened for vector using cross_match software (www.phrap.org), trimmed for vector and quality, and filtered to remove reads shorter than 100 bases. The draft assembly of whole genome Sanger shotgun reads was constructed with Arachne, at an average depth of 12.0 ± 0.15.

Assembled scaffolds were then finished using our Phred/Phrap/Consed pipeline. Initially all low quality regions and gaps were targeted with computationally selected sequencing reactions completed with 4:1 BigDye terminator: dGTP chemistry (Applied Biosystems). These automated rounds included walking on plasmid subclones using custom primers.

Following completion of the automated rounds, a trained finisher manually inspected each assembly. Further reactions were then manually selected to complete the genome. These reactions included additional custom primer walks on plasmid subclones or fosmids. Again the reactions were completed using 4:1 BigDye terminator:dGTP chemistry. Smaller repeats in the sequence were resolved by transposon-hopping 8kb plasmid clones. Fosmid clones were shotgun sequenced and finished to fill large gaps, resolve larger repeats or to resolve chromosome duplications and extend into chromosome telomere regions.

Following completion each assembly was validated by an independent quality assessment. This examination included a visual examination of subclone paired ends and visual inspection of high quality discrepancies and all remaining low quality areas. All available EST resources were also placed on the assembly to ensure completeness.

## 1.4 cDNA library construction and sequencing:

C-169 cells were grown to log phase (1.5 x $10^7$ cells/ml) at 25°C and harvested by centrifugation. The cell pellets were immediately flash frozen in liquid nitrogen, disrupted with glass beads and vortexing in the presence of TRIZOL reagent

(Invitrogen, Carlsbad, CA), and total RNA was isolated according to manufacturer's instructions. The integrity of the sample was evaluated by spectrophotometry and electrophoresis on a denaturing agarose gel. C-169 poly A+ RNA was isolated from total RNA using the Absolutely mRNA Purification kit and manufacturer instructions (Stratagene, La Jolla, CA). cDNA synthesis and cloning was a modified procedure based on the "SuperScript plasmid system with Gateway technology for cDNA synthesis and cloning" (Invitrogen, Carsbad, CA). 1-2 μg of poly A+ RNA, reverse transcriptase SuperScript II (Invitrogen) and oligo dT-NotI primer (5' GACTAGTTCTAGATCGCGAGCGGCCGCCCT15VN 3') were used to synthesize first strand cDNA. Second strand synthesis was performed with *E. coli* DNA ligase, polymerase I, and RNaseH followed by end repair using T4 DNA polymerase. The SalI adaptor (5' TCGACCCACGCGTCCG and 5' CGGACGCGTGGG) was ligated to the cDNA, digested with NotI (New England Biolabs, Ipswich, MA), and subsequently size selected by gel electrophoresis (1.1% agarose). Two size ranges of cDNA were cut out of the gel to generate separate size selected cDNA libraries: 0.6 kb - 2 kb (library codes CPBS and CBWF) and >2 kb (library code CBWC). The cDNA inserts were directionally ligated into the SalI and NotI digested vector pCMVsport6 (Invitrogen). The ligation was transformed into ElectroMAX T1 DH10B cells (Invitrogen).

Library quality was first assessed by randomly selecting 24 clones and PCR amplifying the cDNA inserts with the primers M13-F (5' GTAAAACGACGGCCAGT) and M13-R (5' AGGAAACAGCTATGACCAT) to determine the fraction of clones without inserts. Colonies from each library were plated onto agarose plates (254 mm plates from Teknova, Hollister, CA) at a density of approximately 1,000 colonies per plate. Plates were grown at 37$^{o}$C for 18 h then individual colonies were picked and each used to inoculate a well containing LB media with appropriate antibiotic in a 384 well plate (Nunc, Rochester, NY). Clones in 384 well plates were grown at 37$^{o}$C for 18 h. Plasmid DNA for sequencing was produced by rolling circle amplification (Detter et al., 2002) (Templiphi, GE Healthcare, Piscataway, NJ). Subclone inserts were sequenced from both ends using primers complimentary to the flanking vector sequence (Fw: 5' ATTTAGGTGACACTATAGAA Rv: 5' TAATACGACTCACTATAGGG) with Big Dye terminator chemistry and run on ABI 3730 instruments (Applied Biosystems, Foster City, CA). A total of 28,322 ESTs remained after trimming and filtering.

## 1.5 EST sequence processing and assembly:

A total of 33,024 ESTs including; 16,896 from CBOZ and 16,128 from CBPA were processed through the JGI EST pipeline (ESTs were generated in pairs, a 5' and 3' end read from each cDNA clone). To trim vector and adaptor sequences, common sequence patterns at the ends of ESTs were identified and removed using an internally developed tool. Insertless clones were identified if either of the following criteria were met: >200 bases of vector sequence at the 5' end or less than 100 bases of non-vector sequence remained. ESTs were then trimmed for quality using a sliding window trimmer (window = 11 bases). Once the average quality score in the window was below the threshold (Q15) the EST was split and the longest remaining sequence segment was retained as the trimmed EST. EST sequences with less than 100 bases of high quality sequence were removed. ESTs were evaluated for the presence of poly A or poly T tails, which were removed, and the ESTs were reevaluated for length, removing ESTs with less than 100 bases remaining. ESTs

consisting of more than 50% low complexity sequence were also removed from the final set of "good ESTs". In the case of resequencing the same EST, the longest high quality EST was retained. Sister ESTs (end pair reads) were categorized as follows: if one EST was insertless or a contaminant then by default the second sister was categorized as the same. However, each sister EST was treated separately for complexity and quality scores. Finally, EST sequences were compared to the Genbank nucleotide database in order to identify contaminants; non-desirable ESTs such as those matching non-cellular and rRNA sequences were removed.

For clustering, ESTs were evaluated with malign, a kmer based alignment tool, which clusters ESTs based on sequence overlap (kmer = 16, seed length requirement = 32 alignment ID >= 98%). Clusters of ESTs were further merged based on sister ESTs using double linkage. Double linkage requires that 2 or more matching sister ESTs exist in both clusters to be merged. EST clusters were then each assembled using CAP3 [3] to form consensus sequences. Clusters may have more than one consensus sequence for various reasons to include; the clone has a long insert, clones are splice variants or consensus sequences are erroneously not assembled. Cluster singlets are clusters of one EST, whereas CAP3 singlets are single ESTs which had joined a cluster but during cluster assembly were isolated into a separate singlet consensus sequence. ESTs from each separate cDNA library were clustered and assembled separately and subsequently the entire set of ESTs for all cDNA libraries were clustered and assembled together. For cluster consensus sequence annotation, the consensus sequences were compared to Swissprot using BLASTx and the hits were reported. Clustering and assembly of all 28,322 filtered ESTs resulted in 7,984 consensus sequences.

## 1.6 Phylogenetic analyses of rDNA sequences

The SSU rDNA sequence of C-169 (= NIES 2166) was included into a data set (1746 bp) containing 70 trebouxiophycean representatives. Sequences were aligned according to their secondary structure. This data set was analyzed by PAUP v 4.0b10 [4]. To identify the evolutionary model that best fits the data, log-likelihood values of 56 models were estimated by PAUP and compared by Modeltest [5] using the Akaike Information Criterion. The maximum likelihood tree presented in Additional File 2 - Figure S1B was calculated by PAUP using the GTR+I+G model (settings: base frequencies: A = 0.2492; C = 0.2277; G = 0.2762; T = 0.2469; rate matrix: A-C = 0.9523; A-G = 2.0059; A-T = 0.8932; C-G = 1.3204; C-T = 5.3263; G-T = 1.0000; I = 0.5895; G = 0.5352). To test the robustness of the phylogenetic tree bootstrap analyses (neighbor-joining using the GTR+I+G; 1000 replicates and maximum parsimony, 1000 replicates) were conducted.

To determine to which species C-169 belongs, ITS-1, 5.8S and ITS-2 rDNA sequences of the Elliptochloris-clade (Trebouxiophyceae) were analyzed (Additional File 2 - Figure S1C). Sequences were aligned according to their secondary structures and the resulting data set (27 taxa, 784 bp) was analyzed using the same methods as described above. The evolutionary model used for tree computation was GTR+G (settings: base frequencies: A = 0.2260; C = 0.3122; G = 0.2685; T = 0.1934; rate matrix: A-C = 2.1904; A-G = 3.4773; A-T = 2.2921; C-G = 1.3005; C-T = 5.2156; G-T = 1.0000; G = 0.4036).

# 2 Supplemental Results

## 2.1 Morphology, taxonomy and history of strain C-169

C-169 is a small elongated non-motile unicellular green alga (cell size of 3-9 µm; Additional File 2 - Figure S1) isolated in the polar summer of 1959-60 at Marble Point, Antarctica, from dried algal peat (with associated protozoans and micrometazoans) composed predominantly of *Nostoc commune* and other filamentous blue-green algae [6]. Taxonomic classification of small coccoid green algae such as C-169 has been problematic due to the limited number of morphological traits and high phenotypic variability within species [7]. C-169 was originally classified in the *Chlorella* genus with the species name *Chlorella vulgaris* based on morphological characters [6]. However, as shown in Additional File 2 - Figure S1A the cells are not spherical in shape and the chloroplast does not contain a pyrenoid, two typical characters of *Chlorella vulgaris*. Instead, the morphology of C-169 resembles to that of Coccomyxa species described by Jaag (22) and Acton (21).

Phylogenetic analyses of SSU rDNA sequences confirmed that C-169 belongs to the genus Coccomyxa (Elliptochloris-clade; Trebouxia-lineage) within the Trebouxiophyceae (Additional File 2 - Figure S1B). Trebouxiophyceae is one of the four classes of chlorophytes that also include Chlorophyceae, Ulvophyceae and Mamiellophyceae. Furthermore, analysis of the ITS-1, 5.8S, and ITS-2 rDNA sequences of all *Coccomyxa* species available in Genbank indicated that C-169 clusters together with the authentic strain of *C. subellipsoidea* SAG 216-13 and photobionts isolated from a monophyletic lineage of the basidiolichen *Omphalina* [8] (Additional File 2 - Figure S1C).

Further analysis of the conserved region of the internal transcribed spacer ITS-2, a powerful marker of species delimitation in microalgae [9], indicated that C-169 is almost identical with the strain SAG 216-13, the type strain of *Coccomyxa subellipsoidea* Acton emend. Jaag [10,11]. Comparison of the ITS-2 secondary structures (Additional File 2 - Figure S1D) showed eleven base differences between both strains, but only two hemi-compensatory base changes (changes on only one side of nucleotide pair) could be detected. The level of divergence between the two ITS-2 sequences and the nature of the substitutions (absence of compensatory base changes) are not sufficiently significant according to taxonomic classification standards to consider C-169 as a distinct species from SAG 216-13. The original assignment of this strain as *Chlorella vulgaris* is therefore incorrect. Thus based on solid phylogenetic evidence, we renamed C-169 as *Coccomyxa subellipsoidea* sp. C-169. All nucleotide sequences assigned to the "*Chlorella vulgaris* sp. C-169" strain in public database (total of 67 Kb) aligned with 100% similarity against the genomic sequences reported herein, confirming that *Coccomyxa subellipsoidea* sp. C-169 and *Chlorella vulgaris* sp. C-169 refer to the same organism.

## 2.2 Telomeric repeats and Chromosome count

Coccomyxa sp. C169 was previously shown to have the identical telomeric repeat units as higher plants i.e., TTTAGGG [12]. Of the 29 C-169 scaffolds, 12 have telomeric repeat arrays at both ends and therefore are complete chromosomes ranging from 967 kb to 4.0 Mb in size (Additional File 2 - Figure S2). An additional 16 scaffolds have telomeric repeats at only one end and correspond to terminal segments of chromosomes, likely representing the paired ends of 8 chromosomes. The remaining scaffold, number 28, is an internal chromosome piece with no

telomeric repeat at either end. Based on the terminal telomeric repeat array count, C-169 is predicted to have 20 chromosomes instead of 16 formerly estimated by PFGE [2]. A haploid chromosome number n = 20 is also observed in the green alga *Ostreococcus tauri* [13] and the red alga *Cyanidioschyzon merolae* [14], while the green algae *Chlamydomonas reinhardtii* has only 17 chromosomes [15], C. variabilis has 12 chromosomes [16] and *Ostreococcus lucimarinus* has n = 21 [17].

## 2.3 Carbon Concentrating Mechanism - Conservation Across Algal Species

Several eukaryotic algae, including the widely-studied *Chlamydomonas reinhardtii*, possess a $CO_2$-concentrating mechanism (CCM) that allows accumulation of $CO_2$ and $HCO_3^-$ internally well above that in the external aqueous environment, aiding in photosynthetic efficiency and cell growth [18]. To explore the conservation of this mechanism, eight algal species with sequenced genomes, including *Coccomyxa* sp. C-169, were analyzed computationally for the presence, or absence, of orthologs to various CCM genes present in *C. reinhardtii*.

A total of 22 CCM-related genes in *C. reinhardtii* were selected for analysis. CCM genes (and respective proteins) were searched by BLAST against the proteomes and transcriptome of *Coccomyxa* sp. C169 and eight additional algal species [19,16,20,17,21,13,22,23] using blastp and tblastn, respectively. Alignments generating an expectation value (E-value) of less than $1X10^{-8}$ were marked as candidate genes/proteins. Candidate genes/proteins were then searched reciprocally by BLAST against the *C. reinhardtii* proteome using blastx or blastp. Candidates that aligned with the best bit score to the original CCM protein were considered to be putative CCM orthologs. The findings from this search are summarized in Additional File 1 - Table S9. The proteins CCP1 and CCP2 share a high level of similarity in their amino acid sequences. As a result, putative orthologs for CCP1 were also determined to be putative orthologs for CCP2. This was also found with the proteins LciB, LciC, LciD, and LciE. Therefore, these CCM genes were grouped as 'CCP1,2' and 'LCIB,C,D,E'. This reduced the list of CCM genes from 22 to 18. Of the eight algal species investigated, *Volvox carteri* possessed putative orthologs in 13 of the 18 CCM gene groups. This was followed, in decreasing order of conservation, by *Chlorella variabilis*, C-169, *Micromonas pusilla, Ostreococcus lucimarinus, Ostreococcus tauri, Aureococcus anophagefferens,* and *Thalassiosira pseudonana*.

CIA5/CCM1, the master regulator of the CCM in *Chlamydomonas reinhardtii* [24,25], is suggested to be present in ortholog form in C-169 as well as *Chlorella variabilis* and *Volvox cartieri*. A recent analysis of the CIA5 ortholog in *Volvox carteri* [26] identified conservation of residues found to be critical for zinc binding and/or growth under carbon-limited conditions in the *Chlamydomonas reinhardtii* CIA5. A multiple alignment of *Chlamydomonas reinhardtii* CIA5 with the putative orthologs revealed that these residues are conserved across each of the algal species (Additional File 2 - Figure S8), suggesting CIA5 may retain its role as a regulator of the CCM across these species.

The results of the blast searches and multiple alignments provide an insight into the conservation of CCM genes across algal systems. As mentioned, a number of CCM genes possessed putative orthologs across each algal species. While the presence of these gene orthologs does not validate a functional CCM in each species, it does suggest a conserved importance of these genes in normal cellular function. The conservation of CIA5 across *Coccomyxa* sp. C-169, *Chlorella variabilis*, and *Volvox carteri*, in particular the conservation of residues critical for proper CIA5/CCM1

function, suggests the presence of a functional CCM in each of these species. This hypothesis is supported by data reported earlier for *Volvox carteri* [26]. However, the absence of various CCM gene orthologs in some algal species may indicate an absence of a CCM or the presence of a modified CCM in these algal systems. Further research with these species will be required to verify the presence or absence of additional CCM gene orthologs, as well as the presence or absence of a functional CCM.

# 3 Supporting Tables

**Table S1**: Filtered models (automated annotation) classified by gene prediction method.

| Prediction method | # models |
|---|---|
| total | 9,851 |
| *ab initio* | 5,243 (89%) |
| protein-based | 3,724 (38%) |
| cDNA-based | 884 (9%) |

**Table S2**: Properties of filtered models.

| Property or number | Value |
|---|---|
| Avg. gene length | 3,503 nt |
| Avg. transcript length | 1,474 nt |
| Avg. exon frequency | 8.2 exons per gene |
| # multiexon genes | 9,435 (96%) |
| # genes with similarity to nr protein | 7,839 (80%) |
| # genes in C-169 multigene family | 5,389 (55%) |
| # genes with EST support | 4,982 (51%) |
| # genes with Pfam domain | 5,945 (60%) |
| # genes with signal peptide | 1,886 (19%) |
| # genes with transmembrane domain | 1,737 (18%) |

**Table S3**: Genomic features of sequenced chlorophyte green algae

| Species | Taxonomic class | Nuclear genome size (Mb) | Chromosome number | GC (%) | Repeated sequences (%) | Gene count | Ave. protein length (a.a.) | Ave. Gene density (Kb/gene) | Ave. Intron length (nt) |
|---|---|---|---|---|---|---|---|---|---|
| *Coccomyxa subellipsoidae C-169* | T | 48.8 | 20 | 53 | 7.2 | 9,851 | 425 | 5.0 | 240 |
| *Chlorella variabilis* | T | 46.2 | 12 | 67 | 8.9 | 9,791 | 456 | 4.7 | 174 |
| *Chlamydomonas reinhardtii* | C | 118 | 17 | 64 | 16.7 | 14,516 | 453 | 8.1 | 208 |
| *Volvox carteri* | C | 138 | 14 | 56 | 23.8 | 14,520 | 558 | 9.5 | 294 |
| *Micromonas pusilla* | M | 21.9 | 19 | 65 | 8.8 | 10,575 | 439 | 2.1 | 187 |
| *Micromonas* sp. RCC299 | M | 20.9 | 17 | 64 | 4.6 | 10,056 | 473 | 2.2 | 163 |
| *Ostreococcus tauri* | M | 12.6 | 20 | 59 | 5.1 | 7,892 | 387 | 1.6 | 126 |
| *Ostreococcus lucimarinus* | M | 13.2 | 21 | 60 | 7.7 | 7,651 | 399 | 1.7 | 187 |

T: Trebouxiophyceae ; C : Chlorophyceae ; M : Mamiellophyceae

**Table S4** : Number of unique nucleotides (and fraction) of the organelle genome found integrated into the nuclear genome

| Species | Chloroplast† | Mitochondria† |
|---|---|---|
| *Coccomyxasubellipsoidae* C-169 | 11,312 (6%) | 20,739 (31%) |
| *Chlorellavariabilis* | 517 (0.2%) | 1,329 (1.8%) |
| *Micromonas* RCC299 | 0 | 0 |
| *Micromonas pusilla* | 2,120 | NA‡ |
| *Ostreococcus tauri* | 989 (1.4%) | 699 (0.1%) |
| *Chlamydomonas reinhardtii* | 1,762 (0.6%) | 2,221 (14%) |

†Organelle genome DNA integrated into the nuclear genome was identified by aligning the organelle genome against the nuclear genome with the BLASTN program (E-value<$1^{e}$-20).

‡ The mitochondrial genome sequence of *Micromonas pusilla* was not available in public database.

**Table S5** : Repeated sequences in the C-169 nuclear genome

| Class | Type | Cumulated size (bp) | Percent of total |
|---|---|---|---|
| **LTR Retrotransposon** | | | |
| | Gypsy | 45,400 | 1.3% |
| **Non-LTR Retrotransposon** | | | |
| | LINE-R2 | 8,618 | 0.2% |
| | LINE-RTE | 203,936 | 5.8% |
| | LINE-ZEPP | 571,600 | 16.2% |
| | SINE | 309,261 | 8.8% |
| **DNA transposon** | | | |
| | En-Spm | 58,197 | 1.6% |
| | Tcr1-like | 225,503 | 6.4% |
| **Other repetitive sequences** | | | |
| | Telomeric repeats | 19,758 | 0.6% |
| | rDNA | 52,351 | 1.5% |
| | **duplicated** genes | 412,465 | 11.7% |
| | unknown sequences | 1,620,542 | 45.9% |
| TOTAL | | 3,527,634 | 100.0% |

**Table S6**: PFAM protein families with biased distribution in Coccomyxa

| PFAM ID | PFAM name | *C. subellipsoidae* C-169 | *Chlorella variabilis* | *C. reinhardtii* | *V. carteri* | *Micromonas pusilla* | *Micromonas sp.* RCC299 | *O. lucimarinus* | *O. tauri* | *Ostreococcus sp.* RCC809 | p-value ‡ | Putative function |
|---|---|---|---|---|---|---|---|---|---|---|---|---|
| **Transport** | | | | | | | | | | | | |
| PF00324 | AA_permease | 15 | 5 | 4 | 6 | 1 | 3 | 1 | 1 | 1 | 3.77E-06 | Amino acid permease family |
| PF01490 | Aa_trans | 21 | 37 | 8 | 6 | 7 | 8 | 6 | 6 | 4 | 2.49E-14 | Transmembrane amino acid transporter family |
| PF11744 | ALMT | 6 | 2 | 0 | 0 | 0 | 1 | 0 | 0 | 0 | 9.31E-05 | Aluminium activated malate transporter family |
| **Lipid metabolism** | | | | | | | | | | | | |
| PF08392 | FAE1_CUT1_RppA | 11 | 2 | 5 | 4 | 0 | 0 | 0 | 0 | 0 | 2.47E-07 | Fatty acid elongase family |
| PF08541 | ACP_syn_III_C | 11 | 1 | 2 | 2 | 1 | 1 | 1 | 1 | 1 | 1.22E-05 | 3-Oxoacyl-[acyl-carrier-protein (ACP)] synthase domain of fatty acid elongase |
| PF07993 | NAD_binding_4 | 7 | 0 | 0 | 1 | 0 | 1 | 0 | 0 | 1 | 1.25E-05 | Fatty acyl-CoA ligase family |
| PF01764 | Lipase_3 | 30 | 29 | 14 | 12 | 8 | 14 | 8 | 9 | 8 | 1.44E-06 | Lipase (class 3) family |
| PF08659 | KR | 9 | 1 | 1 | 1 | 2 | 2 | 1 | 2 | 0 | 7.22E-04 | Keto reductase domain of fatty acid synthase type I and polyketide synthase |
| **Glycan and cell wall metabolism** | | | | | | | | | | | | |
| PF00150 | Cellulase | 22 | 7 | 4 | 3 | 2 | 3 | 2 | 2 | 3 | 1.68E-10 | Cellulase (glycosyl hydrolase family 5) |
| PF00759 | Glyco_hydro_9 | 9 | 6 | 3 | 3 | 0 | 0 | 0 | 0 | 0 | 1.22E-05 | Glycosyl hydrolase domain of bifunctional cellulose synthase - cellulase proteins |
| PF01357 | Pollen_allerg_1 | 7 | 4 | 0 | 0 | 0 | 0 | 0 | 0 | 0 | 1.25E-06 | Pollen allergen domain of expansin |
| PF00201 | UDPGT | 7 | 4 | 1 | 1 | 0 | 0 | 0 | 0 | 0 | 5.25E-05 | UDP-glucoronosyl and UDP-glucosyl transferase |
| PF02485 | Branch | 11 | 10 | 0 | 0 | 1 | 1 | 0 | 0 | 0 | 6.76E-11 | beta-1,6-N-acetylglucosaminyltransferase family |
| **Miscellaneous** | | | | | | | | | | | | |
| PF00106 | adh_short | 70 | 47 | 36 | 39 | 35 | 38 | 23 | 21 | 22 | 2.63E-08 | short chain dehydrogenase family |

‡ P-value associated to the null hypothesis that the number of proteins assigned to the PFAM category is evenly distributed among the 9 algae. The P-value was calculated using the Chi square test.

**Table S7**: Fatty acid synthase type II subunit proteins in *C. ellipsoidae* C-169

| Fatty acid synthase type II Subunit name | organelle | Accession number of reference | reference organism | candidate orthologue in C-169 (protein id) † | Number of EST for C-169 gene | function |
|---|---|---|---|---|---|---|
| AT | plastid | AT2G30200 | *A. thaliana* | 27235 | 15 | malonyl CoA:ACP acyltransferase |
| | mitochondria | Q8IVS2 | *H. sapiens* | 16677 | 0 | |
| KAS | plastid | AT5G46290 (KAS1) | *A. thaliana* | 54810 | 25 | β-ketoacyl-ACP synthase |
| | plastid | AT1G74960 (KAS2) | *A. thaliana* | 54967 | 2 | |
| | plastid | AT1G62640 (KAS3) | *A. thaliana* | 54940 | 1 | |
| | mitochondria | CAB58180 | *S. cerevisiae* | 11277 | 0 | |
| DH | plastid | AT2G22230 | *A. thaliana* | 53697 | 9 | 3-hydroxyacyl-CoA dehydrogenase |
| | mitochondria | NP_001171634 | *H. sapiens* | 37630 | 0 | |
| KR | plastid | AT1G24360 | *A. thaliana* | 33281 | 13 | 3-oxoacyl-ACP reductase |
| | mitochondria | CAA81892 | *S. cerevisiae* | 66711 | 10 | |
| ER | plastid | AT2G05990 | *A. thaliana* | 65820 | 3 | trans-2-enoyl-CoA reductase |
| | mitochondria | EEU08119 | *S. cerevisiae* | Not found | | |
| ACP | plastid | AT1G54580 | *A. thaliana* | 52715 | 13 | Acyl carrier protein |
| | mitochondria | AAX69898 | *T. brucei* | 53954 | 19 | |

† : identified by reciprocal best blastp hit with E-value < 1e-5.

**Table S8** : C-169 protein genes that have no homologue in the other sequenced chlorophytes

| Protein id | Putative function | Taxonomic classification of closest phylognetic clade* |
|---|---|---|
| ***Nitrogen metabolism*** | | |
| 68344 | 2-nitropropane dioxygenase | Fungi |
| 28301, 29543, 48328 | Ureide permease | Streptophyta |
| ***Amino acid metabolism*** | | |
| 39859 | Kynureninase | Bacteria |
| ***Carbohydrate metabolism*** | | |
| 61318, 66312 | Alanine racemase | Bacteria |
| 45472, 41680, 66875, 43872, 56741, 56745, 56744 | Alpha-1,3-galactosidase B | Lentisphaerae |
| 57209 | Alpha-L-arabinofuranosidase-like protein | Bacteria |
| 42818, 45437 | Beta-glucanase | Fungi |
| 44687, 58627, 64481 | Beta-glucuronidase | Actinobacteria |
| 64481 | Bifunctional xylanase/deacetylase | Proteobacteria |
| 40396, 46570, 61296, 40400, 59785, 63419, 63418 | Cellulase | Actinobacteria |
| 45133 | Endoglucanase E | Firmicutes |
| 68001 | Endoglycosylceramidase | Proteobacteria |
| 65352 | Exordium like protein | Streptophyta |
| 9432, 9502, 48268 | Exostosin family protein | Alveolata |
| 39799, 8290 | Gluconolactonase | Proteobacteria |
| 8290, 39799 | Gluconolactonase | Fungi |
| 26821 | Glycosylasparaginase-like protein | Streptophyta |
| 41573 | Glycosylhydrolase 18 | Proteobacteria |
| 56271, 56272, 55028 | Glycosyltransferase 1 | Proteobacteria |
| 45597, 42571, 58627, 44686, 44687 | Glycosyltransferase 2 | Bacteria |
| 56271, 56272, 55028 | Glycosyltransferase 8 | Metazoa |
| 12587 | Lysozyme | Proteobacteria |
| 44565 | N-acetyl glucosaminyl transferase | Proteobacteria |
| 12402 | N-acetyl-glucosamine-1-phosphate uridyltransferase | Proteobacteria |
| 57018 | Sucrose phosphorylase | Bacteria |
| 49413 | Xyloglucan fucosyltransferase | Streptophyta |
| ***Glyoxylate and dicarboxylate metabolism*** | | |
| 42471, 64000 | Oxalate decarboxylase OxdD | Cyanobacteria |
| 52070 | Germin-like oxalate oxidase | Streptophyta |
| ***Defense and detoxication*** | | |
| 28192 | Arsenic resistance protein ArsH | Fungi |
| 14170 | Arsenite transporter protein arsB-like | Streptophyta |
| 39956, 46827, 67338, 45789 | Beta-lactamase | Firmicutes |
| 45842 | Chalcone synthase-like protein | Streptophyta |
| 52516 | DOPA-dioxygenase | Alphaproteobacteria |
| 63999, 66435 | Epoxide hydrolase | Actinobacteria |

| Protein id | Putative function | Taxonomic classification of closest phylognetic clade* |
|---|---|---|
| 61356, 57614 | Glutathione-dependent formaldehyde-activating enzyme | Proteobacteria |
| 64436 | Metallo-beta-lactamase-like protein | Chloroflexi |
| 63635, 63636 | Quinone reductase | Actinobacteria |
| 20637 | RNA-dependent RNA polymerase | Streptophyta |
| ***DNA/RNA metabolism*** | | |
| 42033 | endonuclease 4 | Metazoa |
| 58564 | endonuclease | Firmicutes |
| ***Lipid metabolism/signalling*** | | |
| 38692 | Phospholipase D | Streptophyta |
| 41733 | lipid kinase | Proteobacteria |
| ***Translation*** | | |
| 54652 | Elongation factor 1-alpha | Streptophyta |
| ***Transport*** | | |
| 61602 | cation efflux protein | Stramenopiles |
| 42251 | chloride transporter, ClC family | Stramenopiles |
| 41263, 64990 | Major Facilitator Superfamily transporter | Streptophyta |
| 65924, 68148 | Major Facilitator Superfamily transporter | Bacteria |
| 64250 | Na+/solute symporter euk | Stramenopiles |
| 46890 | Transmembrane channel protein | Streptophyta |
| ***Miscellaneous*** | | |
| 59480 | amidohydrolase | Actinobacteria |
| 42526 | Bacteriorhodopsin-like light-driven proton pump protein | Glaucophyta |
| 40283 | bZIP transcription factor | Streptophyta |
| 61025 | FAD linked oxidase domain-containing protein | Stramenopiles |
| 63892 | flavin-binding monooxygenase-like protein | Fungi |
| 21900, 28854 | Flavin-nucleotide-binding protein | Bacteria |
| 54197 | glutathione S-transferase-like protein | Cyanobacteria |
| 11790 | LysR-type transcriptional regulator | Bacteria |
| 47584 | NAD dependent epimerase/dehydratase family protein | Fungi |
| 48058 | Oxidoreductase | Stramenopiles |
| 33070 | Retrovirus-related polyprotein | Metazoa |
| 39290, 64345 | Salicylate esterase | Actinobacteria |
| 66539 | SAM-dependant methyltransferase | Streptophyta |
| 58196 | serine peptidase | Proteobacteria |
| 66251 | sugar fermentation stimulation protein | Firmicutes |
| 65285 | TCTP protein | Streptophyta |
| 68066 | X-Pro dipeptidyl-peptidase | Stramenopiles |
| 52998 | Zinc finger protein | Streptophyta |
| 9061 | Unknown | Fungi |
| 10993 | Unknown | Streptophyta |
| 17537 | Unknown | Streptophyta |
| 17931 | Unknown | Streptophyta |
| 32414 | Unknown | Fungi |
| 33164 | Unknown | Eukaryota |
| 33282 | Unknown | Streptophyta |

| Protein id | Putative function | Taxonomic classification of closest phylognetic clade* |
|---|---|---|
| 33454 | Unknown | Streptophyta |
| 34634 | Unknown | Eukaryota |
| 37587 | Unknown | Streptophyta |
| 39032 | Unknown | Eukaryota |
| 39310 | Unknown | Bacteria |
| 39797 | Unknown | Streptophyta |
| 39880 | Unknown | Streptophyta |
| 40341 | Unknown | Eukaryota |
| 41138 | Unknown | Stramenopiles |
| 41553 | Unknown | Bacteria |
| 41585 | Unknown | Streptophyta |
| 41610 | Unknown | Eukaryota |
| 42235 | Unknown | Streptophyta |
| 42320 | Unknown | Streptophyta |
| 42321 | Unknown | Streptophyta |
| 42447 | Unknown | Streptophyta |
| 42817 | Unknown | Streptophyta |
| 42889 | Unknown | Eukaryota |
| 44554 | Unknown | Bacteria |
| 44822 | Unknown | Eukaryota |
| 45133 | Unknown | Eukaryota |
| 45326 | Unknown | Eukaryota |
| 45353 | Unknown | Streptophyta |
| 45928 | Unknown | Bacteria |
| 47036 | Unknown | Bacteria |
| 47264 | Unknown | Eukaryota |
| 47556 | Unknown | Eukaryota |
| 47613 | Unknown | Eukaryota |
| 47770 | Unknown | Streptophyta |
| 47982 | Unknown | Streptophyta |
| 48284 | Unknown | Streptophyta |
| 48684 | Unknown | Streptophyta |
| 48716 | Unknown | Streptophyta |
| 52214 | Unknown | Eukaryota |
| 52224 | Unknown | Bacteria |
| 52901 | Unknown | Streptophyta |
| 52943 | Unknown | Bacteria |
| 53472 | Unknown | Bacteria |
| 53620 | Unknown | Eukaryota |
| 53747 | Unknown | Streptophyta |
| 54113 | Unknown | Eukaryota |
| 54324 | Unknown | Streptophyta |
| 54388 | Unknown | Eukaryota |
| 54594 | Unknown | Stramenopiles |
| 55252 | Unknown | Streptophyta |
| 55290 | Unknown | Eukaryota |
| 55477 | Unknown | Streptophyta |
| 55651 | Unknown | Eukaryota |
| 55952 | Unknown | Eukaryota |
| 55993 | Unknown | Streptophyta |

| Protein id | Putative function | Taxonomic classification of closest phylognetic clade* |
|---|---|---|
| 56281 | Unknown | Bacteria |
| 56329 | Unknown | Streptophyta |
| 56344 | Unknown | Heterolobosea |
| 56518 | Unknown | Streptophyta |
| 57808 | Unknown | Streptophyta |
| 58082 | Unknown | Streptophyta |
| 58372 | Unknown | Eukaryota |
| 58484 | Unknown | Bacteria |
| 58494 | Unknown | Bacteria |
| 58606 | Unknown | Eukaryota |
| 59402 | Unknown | Eukaryota |
| 59427 | Unknown | Streptophyta |
| 59565 | Unknown | Bacteria |
| 59756 | Unknown | Bacteria |
| 59807 | Unknown | Streptophyta |
| 60079 | Unknown | Streptophyta |
| 60297 | Unknown | Streptophyta |
| 60661 | Unknown | Eukaryota |
| 60683 | Unknown | Streptophyta |
| 61117 | Unknown | Eukaryota |
| 61428 | Unknown | Metazoa |
| 61835 | Unknown | Eukaryota |
| 61842 | Unknown | Bacteria |
| 61953 | Unknown | Eukaryota |
| 62040 | Unknown | Streptophyta |
| 62533 | Unknown | Streptophyta |
| 62534 | Unknown | Streptophyta |
| 62698 | Unknown | Streptophyta |
| 62796 | Unknown | Bacteria |
| 62797 | Unknown | Bacteria |
| 62901 | Unknown | Eukaryota |
| 62943 | Unknown | Firmicutes |
| 63118 | Unknown | Eukaryota |
| 63243 | Unknown | Streptophyta |
| 63277 | Unknown | Eukaryota |
| 63577 | Unknown | Eukaryota |
| 64828 | Unknown | Bacteria |
| 64923 | Unknown | Eukaryota |
| 65346 | Unknown | Streptophyta |
| 66025 | Unknown | Eukaryota |
| 66031 | Unknown | Streptophyta |
| 66057 | Unknown | Eukaryota |
| 66327 | Unknown | Streptophyta |
| 66380 | Unknown | Streptophyta |
| 66759 | Unknown | Eukaryota |
| 66850 | Unknown | Streptophyta |
| 67529 | Unknown | Eukaryota |
| 67562 | Unknown | Eukaryota |
| 68254 | Unknown | Bacteria |
| 68290 | Unknown | Streptophyta |

| Protein id | Putative function | Taxonomic classification of closest phylognetic clade* |
|---|---|---|
| 68334 | Unknown | Bacteria |
| 68343 | Unknown | Eukaryota |
| 21900, 28854 | Unknown | Bacteria |
| 33968, 32478 | Unknown | Eukaryota |
| 39504, 64411 | Unknown | Streptophyta |
| 44028, 57844 | Unknown | Eukaryota |
| 53569, 53568 | Unknown | Eukaryota |
| 56274, 66698 | Unknown | Bacteria |
| 58094, 44273, 58092 | Unknown | Bacteria |
| 58584, 44361 | Unknown | Bacteria |
| 58779, 40319 | Unknown | Bacteria |
| 58841, 63391 | Unknown | Streptophyta |
| 60694, 39973 | Unknown | Eukaryota |
| 60700, 65392 | Unknown | Streptophyta |
| 61303, 64687, 68213, 45656 | Unknown | phycoDNAvirus |
| 62533, 62534 | Unknown | Streptophyta |
| 62874, 62877 | Unknown | Bacteria |
| 64990, 41263 | Unknown | Streptophyta |
| 66858, 66960, 43154, 43152, 43198, 55863, 56731, 44250 | Unknown | Streptophyta |
| 67155, 40205 | Unknown | Eukaryota |

* determined using the BLAST-EXPLORER webtool (Dereeper et al., 2010) that combines BLASTP searches with a phylogenetic reconstruction of the query and hit sequences.

**Table S9**: Putative ortholog chart for CCM genes across nine algal species.

<table>
<tr><th>genes</th><th>C. reinhardtii Genbank accession number</th><th>Coccomyxa sp. C169</th><th>Chlorella sp. NC64A</th><th>Volvox carteri</th><th>Micromonas pusilla</th><th>Ostreococcus lucimarinus</th><th>Ostreococcus tauri</th><th>Thalassiosira pseudonana</th><th>Aureococcus anophagefferens</th></tr>
<tr><td>CAH1</td><td>XP_001692291</td><td>jgi|Coc_C169_1|67822<br>jgi|Coc_C169_1|48539</td><td>jgi|ChlNC64A_1|59546<br>jgi|ChlNC64A_1|59547</td><td>jgi|Volca1|109791<br>jgi|Volca1|61206<br>jgi|Volca1|71391</td><td>jgi|MicpuC2|38680</td><td></td><td></td><td></td><td></td></tr>
<tr><td>CAH3</td><td>XP_001696744</td><td></td><td>jgi|ChlNC64A_1|11032</td><td>jgi|Volca1|109995</td><td></td><td></td><td></td><td></td><td></td></tr>
<tr><td>CCP1,2</td><td>XP_001692197<br>XP_001692288</td><td></td><td></td><td>jgi|Volca1|61165</td><td></td><td></td><td></td><td>jgi|Thaps3|40010</td><td>jgi|Auran1|28315</td></tr>
<tr><td>CIA5</td><td>XP_001699846</td><td>jgi|Coc_C169_1|68177</td><td>jgi|ChlNC64A_1|137649</td><td>jgi|Volca1|99741</td><td></td><td></td><td></td><td></td><td></td></tr>
<tr><td>ELI4</td><td>XP_001694984</td><td></td><td>jgi|ChlNC64A_1|142108</td><td>jgi|Volca1|107717</td><td></td><td></td><td></td><td></td><td></td></tr>
<tr><td>GGPS</td><td>XP_001703169</td><td>jgi|Coc_C169_1|12737</td><td>jgi|ChlNC64A_1|31906</td><td>jgi|Volca1|109214</td><td>jgi|MicpuC2|24186</td><td>jgi|Ost9901_3|17563</td><td>jgi|Ostta4|26626</td><td>jgi|Thaps3|268480</td><td>jgi|Auran1|52464</td></tr>
<tr><td>HLA3</td><td>XP_001700040</td><td></td><td>jgi|ChlNC64A_1|138483<br>jgi|ChlNC64A_1|58775</td><td>jgi|Volca1|104778</td><td></td><td></td><td></td><td></td><td></td></tr>
<tr><td>LCI1</td><td>XP_001703387</td><td></td><td></td><td></td><td></td><td></td><td></td><td></td><td></td></tr>
<tr><td>LCI19</td><td>XP_001698820</td><td></td><td></td><td>jgi|Volca1|65267</td><td></td><td></td><td></td><td></td><td></td></tr>
<tr><td>LCI24</td><td>XP_001703408</td><td>jgi|Coc_C169_1|67162</td><td></td><td></td><td></td><td></td><td></td><td></td><td></td></tr>
<tr><td>LCI6</td><td>XP_001694024</td><td></td><td></td><td></td><td></td><td></td><td></td><td></td><td></td></tr>
<tr><td>LCI8</td><td>XP_001694990</td><td>jgi|Coc_C169_1|21827</td><td>jgi|ChlNC64A_1|137645<br>jgi|ChlNC64A_1|7287</td><td>jgi|Volca1|57336</td><td>jgi|MicpuC2|45566</td><td>jgi|Ost9901_3|1828</td><td>jgi|Ostta4|1745</td><td>jgi|Thaps3|263866<br>jgi|Thaps3|30701<br>jgi|Thaps3|36764<br>jgi|Thaps3|38656</td><td>jgi|Auran1|11234<br>jgi|Auran1|71410</td></tr>
<tr><td>LCIA</td><td>XP_001691213</td><td></td><td>jgi|ChlNC64A_1|34412</td><td>jgi|Volca1|75029</td><td>jgi|MicpuC2|26630</td><td>jgi|Ost9901_3|25116</td><td>jgi|Ostta4|19784</td><td></td><td>jgi|Auran1|15503<br>jgi|Auran1|53005</td></tr>
<tr><td>LCIB,C, D,E</td><td>XP_001698344<br>XP_001691223<br>XP_001692192<br>XP_001692293</td><td>jgi|Coc_C169_1|16853<br>jgi|Coc_C169_1|21111</td><td>jgi|ChlNC64A_1|145145<br>jgi|ChlNC64A_1|33527<br>jgi|ChlNC64A_1|53648<br>jgi|ChlNC64A_1|58491<br>jgi|ChlNC64A_1|8957<br>jgi|ChlNC64A_1|8980</td><td>jgi|Volca1|64669<br>jgi|Volca1|81494<br>jgi|Volca1|84075</td><td>jgi|MicpuC2|46507</td><td>jgi|Ost9901_3|42672</td><td>jgi|Ostta4|34478</td><td></td><td></td></tr>
<tr><td>LCR1</td><td>BAD13491</td><td></td><td></td><td></td><td></td><td></td><td></td><td></td><td></td></tr>
<tr><td>MCA1</td><td></td><td>jgi|Coc_C169_1|68079</td><td>jgi|ChlNC64A_1|140648</td><td>jgi|Volca1|57174</td><td>jgi|MicpuC2|49424</td><td>jgi|Ost9901_3|14352</td><td></td><td></td><td></td></tr>
<tr><td>NDA3</td><td>XP_001702271</td><td>jgi|Coc_C169_1|66639</td><td>jgi|ChlNC64A_1|143182</td><td>jgi|Volca1|105834<br>jgi|Volca1|63330</td><td></td><td></td><td></td><td>jgi|Thaps3|36673<br>jgi|Thaps3|38312<br>jgi|Thaps3|6361<br>jgi|Thaps3|687</td><td>jgi|Auran1|10731</td></tr>
<tr><td>OHP1</td><td>XP_001696654</td><td>jgi|Coc_C169_1|66513</td><td>jgi|ChlNC64A_1|58105</td><td></td><td>jgi|MicpuC2|56686</td><td>jgi|Ost9901_3|9364</td><td>jgi|Ostta4|27642</td><td></td><td></td></tr>
<tr><td>STA2</td><td>XP_001697117</td><td>jgi|Coc_C169_1|52411<br>jgi|Coc_C169_1|54144</td><td>jgi|ChlNC64A_1|145120<br>jgi|ChlNC64A_1|58262</td><td>jgi|Volca1|72820</td><td>jgi|MicpuC2|49698</td><td>jgi|Ost9901_3|49880</td><td>jgi|Ostta4|28387</td><td></td><td></td></tr>
</table>

**Table 10** : Genbank accession numbers of proteins that exist in all sequenced chlorophytes except C-169

| *O. lucimarinus* | *O. tauri* | *M. pusilla* | *Micromonas RCC299* | *C. reinhardtii* | *V. carteri* | *C. variabilis* | putative function |
|---|---|---|---|---|---|---|---|
| ***Carbohydrate metabolism*** | | | | | | | |
| XP_001420094 | XP_003081518 | XP_003062733 | XP_002503860 | XP_001701581 | XP_002957310 | EFN53992 | Dolichyldiphosphatase |
| XP_001415524 | XP_003074347 | XP_003056551 | XP_002507173 | XP_001699060 | XP_002945676 | EFN53048 | mannosyltransferase |
| XP_001420039 | TBLASTN* | XP_003055664 | XP_002503466 | XP_001700037 | XP_002958711 | EFN55058 | Carbohydrate kinase |
| XP_001416304 | XP_003075283 | XP_003060034 | XP_002505720 | XP_001702572 | XP_002955807 | EFN51244 | Pyruvate, phosphate dikinase |
| ***Glycosyl phosphatidyl inositol (GPI) pathway*** | | | | | | | |
| XP_001419518 | XP_003081134 | XP_003056756 | XP_002506136 | XP_001699434 | XP_002958871 | EFN58972 | GPI transamidase Gaa1 subunit |
| XP_001418008 | XP_003079362 | XP_003056932 | XP_002502505 | XP_001690356 | XP_002946567 | EFN58313 | GPI transamidase Gpi16 subunit |
| XP_001421205 | XP_003083197 | XP_003058725 | XP_002503021 | XP_001697551 | XP_002950169 | EFN57492 | GPI transamidase Gpi8 subunit |
| XP_001418580 | XP_003080108 | XP_003055730 | XP_002499477 | XP_001693624 | XP_002948828 | EFN59535 | GPI anchor biosynthesis protein Gwt |
| ***Transport*** | | | | | | | |
| XP_001422192 | XP_003084074 | XP_003064546 | XP_002501127 | XP_001690975 | XP_002949385 | EFN59045 | sodium/sulfate co-transporter |
| XP_001416507 | XP_003075124 | XP_003064656 | XP_002503145 | XP_001702048 | XP_002953275 | EFN55998 | membrane transporter |
| XP_001418299 | XP_003080018 | XP_003062006 | XP_002508125 | XP_001703253 | XP_002946878 | EFN59197 | MFS family transporter |
| XP_001418450 | XP_003079839 | XP_003062066 | XP_002508803 | XP_001690493 | XP_002958358 | EFN55365 | voltage-gated ion channel |
| XP_001419813 | XP_003081306 | XP_003057138 | XP_002506009 | XP_001690895 | XP_002948329 | EFN52811 | maltose exporter protein |
| ***Amino acid biosynthesis*** | | | | | | | |
| XP_001417046 | XP_003078364 | XP_003055460 | XP_002503674 | XP_001696420 | XP_002947125 | EFN52253 | cobalamin-dependent methionine synthase |
| ***Photosynthesis*** | | | | | | | |
| XP_001418138 | XP_003079643 | XP_003059402 | XP_002500817 | XP_001701700 | XP_002953671 | EFN52626 | PS1 reaction center subunit N |
| ***Miscellaneous*** | | | | | | | |
| XP_001417859 | XP_003079552 | XP_003057304 | XP_002508491 | XP_001693805 | XP_002948881 | EFN59375 | Amino acid kinase family protein |
| XP_001420178 | XP_003081705 | XP_003055049 | XP_002503895 | XP_001695478 | XP_002955339 | EFN59998 | DNA-binding protein HU |
| XP_001417847 | XP_003079531 | XP_003059518 | XP_002502234 | XP_001699167 | XP_002958353 | EFN58161 | Iron-containing alcohol dehydrogenase |
| XP_001422472 | XP_003082844 | XP_003061519 | XP_002509237 | XP_001696863 | XP_002953402 | EFN54560 | NUDIX (nucleoside diphosphate linked moiety X) family hydrolase |
| XP_001420876 | XP_003082468 | XP_003063330 | XP_002504562 | XP_001689978 | XP_002956632 | EFN54272 | oligoendopeptidase, M3 family |
| XP_001416237 | XP_003075116 | XP_003060209 | XP_002504149 | XP_001700121 | XP_002957481 | EFN52607 | UNKNOWN |
| XP_001417197 | XP_003078680 | XP_003060348 | XP_002505269 | XP_001695550 | XP_002950102 | EFN56280 | UNKNOWN |
| XP_001418863 | XP_003080209 | XP_003055175 | XP_002499987 | XP_001702217 | XP_002953126 | EFN59421 | UNKNOWN |
| XP_001421045 | XP_003082522 | XP_003064720 | XP_002505883 | XP_001692402 | XP_002951729 | EFN52697 | UNKNOWN |
| XP_001421490 | XP_003083335 | XP_003060565 | XP_002505047 | XP_001693986 | XP_002956351 | EFN57285 | UNKNOWN |
| XP_001421359 | XP_003083438 | XP_003061015 | XP_002502902 | XP_001693277 | XP_002957239 | EFN58853 | UNKNOWN |
| XP_001417059 | XP_003078383 | XP_003054805 | XP_002507062 | XP_001700987 | XP_002954318 | EFN52619 | UNKNOWN |
| XP_001419244 | XP_00308098 | XP_003058628 | XP_002508018 | XP_001692128 | XP_002952100 | EFN59672 | UNKNOWN |
| XP_001417781 | XP_003079390 | XP_003057357 | XP_002502176 | XP_001693981 | XP_002956347 | EFN55956 | UNKNOWN |

* the protein gene absent from the official genome annotation but it was identified by TBLASTN alignment against the genome sequence using the *O. lucimarinus* protein as query

# 4 Supporting Figure Legends

**Figure S1**: A taxonomy study of C-169

A: Morphology of C-169 (1) compared to the authentic strain of *Coccomyxa subellipsoidea* SAG 216-13 (2), the original figures published by Acton (3) and Jaag (4) reporting the first formal descriptions of *C. subellipsoidea*.

B: Molecular phylogeny of the Trebouxiophyceae based on SSU rDNA sequence comparisons. The phylogenetic tree shown was reconstructed using the maximum likelihood method based on a data set of 1746 aligned positions of 70 taxa. Bootstrap values (> 70%) of the neighbour-joining (using the GTR+I+G model, 1000 replicates), and maximum parsimony (1000 replicates) were marked with branches in bold in the tree. Strain and accession numbers are given after the species name. The clade designation follows Pröschold et al. (27). The *Oocystis*-clade was used as out-group.

C: Molecular phylogeny of the *Elliptochloris*-clade based on ITS-1, 5.8S, and ITS-2 rDNA sequence comparisons. The phylogenetic tree shown was inferred by maximum likelihood method based on a data set of 784 aligned positions of 27 taxa. Bootstrap values (> 70%) of the neighbour-joining (using the GTR+G model, 1000 replicates), and maximum parsimony (1000 replicates) were marked with branches in bold in the tree. Strain and accession numbers are given after the species name. *Elliptochloris bilobata* was used as out-group.

D: Comparison of the ITS-2 secondary structure of strains belonging to *Coccomyxa subellipsoidea* Acton emend Jaag. The C-169 ITS-2 sequence is presented with differences with that of SAG 216-13, the authentic strain of *C. subellipsoidea*, highlighted. Single base changes are encircled. The hemi-compensatory base changes are marked with an asterisk. The numbering of the helices followed the designation described by Coleman & Mai (1997). The arrows in Helix II and III mark the RNA processing sites, which are pyrimidine-pyrimidine mismatch in Helix II (usually a UxU mismatch after the fifth or sixth base pairs; in our case a bulge of three unpaired base pairs after the fourth base pair) and the GGU at the 5' site of Helix III (present in all Viridiplantae: Green algae and higher plants). The two arrows at the 5.8S-LSU stem mark the cutting site, where the ITS-2 is cut during the RNA processing.

E: A maximum likelihood phylogenetic tree of sequenced chlorophytes. The phylogenetic tree was computed using the WAG+G+I model of amino acid substitution from a concatenated alignment of 1,253 ubiquitous orthologous proteins totalizing 263,131 gap-free sites. All interior nodes received 100% bootstrap support (aLRT).

**Figure S2**. General characteristics of the *C. subellipsoidea* C-169 genome assembly

This figure represents the 29 scaffolds of the C-169 genome assembly. GC percentage, exon density, EST density, and repeat density were calculated in 40-kb sliding windows with a step of 5 kb. Density was calculated as the percentage of nucleotide in the window covered by the relevant feature (i.e., exon, EST, or repeat sequence). Scaffolds with numbers circled in red correspond to chromosomes that were fully reconstructed from sequence assembly. Scaffolds with numbers circled in blue form complete chromosomes joined from hybridization evidence. Blue and red triangles represent telomeric repeat arrays and ZEPP retrotransposon clusters.

**Figure S3**: Synteny correlation and conservation of gene order are inversely correlated with evolutionary distance

A: Protein distance versus number of conserved pairs of adjacent orthologues (CPAO) calculated between pairs of organisms in the chlorophyte phylogenetic tree shown in fig. S4C. For nodes with more than 2 leaves, we plotted the averages of protein distances and numbers of CPAO between all possible pairs of organisms sharing the same last common ancestor. Pairwise protein distances were calculated from a concatenated alignment of 1,253 orthologous proteins shared by all sequenced chlorophytes (totalizing 263,131 gap-free sites). Pairwise distances were calculated using the maximum likelihood method implemented in the PUZZLE program and the WAG+G substitution model. An exponential regression line is shown in black, with equation and correlation coefficient indicated on the top right side of the plot.

B: Same as A with synteny correlation coefficients plotted on the y-axis.

C: Phylogenetic tree of the sequenced chlorophytes with nodes numbered and shaded according to A and B.

**Figure S4**: Maximum likelihood phylogenetic tree of fatty acid desaturase proteins specific to C-169

The multiple-sequence alignment contained 240 gap-free sites. The phylogenetic tree was reconstructed using the WAG+I+G model of substitutions and the PHYML program. Approximate likelihood ratio test values for nodes are indicated beside branches. Genbank identification numbers are indicated after species names, except for C-169 proteins where JGI protein identification number are given.

**Figure S5**: Maximum likelihood phylogenetic tree of non-ribosomal protein synthase terminal domains.

The phylogenetic tree was constructed using the WAG+G+I substitution model and the PHYML program. The multiple-sequence alignment contained 494 gap-free columns. Approximate likelihood ratio test (aLRT) values for branch support are indicated beside branches when aLRT >50. Genbank identification (gi) numbers and JGI protein ids (C-169) are indicated between brackets. The functional domain architecture of proteins is shown on the right. Domain names are as follows: ACP: Acyl Carrier protein; KAS: ketoacyl-ACP synthase; AT: acyl transferase; DH: hydroxyacyl-ACP dehydrase; MT: methyltransferase; ER: enoyl-ACP reductase; KR: ketoacyl-ACP reductase; NRPS: non-ribosomal protein synthase terminal domain; LuxE: LuxE family peptide synthase; Ox: oxydoreductase; Cata: catalase.

**Figure S6**: Maximum likelihood phylogenetic tree of cellulose synthase-like protein domains

The multiple-sequence alignment contained 340 gap-free sites. The phylogenetic tree was reconstructed using the WAG+I+G substitution model and the PHYML program. Approximate likelihood ratio test (aLRT) values >50 are indicated beside branches. Branches with aLRT<50 were collapsed. Genbank and JGI ids of sequences are given between curly brackets, respectively. The *Chlorella variabilis* protein missing from the current version of the genome annotation was identified on the scaffold 12 by a TBLASTN search using the C-169 protein as query. A scheme of the general protein domain architecture is given for each major sequence clade; CesA: Cellulose Synthase-like domain (cd06421), GH6 and GH9: Glycosyl hydrolase

type 6 (PF01341) and type 9 (PF00759) respectively. The algal cellulose synthase-like domains featured motifs common to UDP-dependent, polymerizing β-glycosyltransferases in the GT-2 family, wherein the conserved "D, DxD" and "D, QxxRW" residues form the binding sites for the donor UDP and acceptor sugar, respectively [27]. In contrast to the other proteins in the tree, the algal proteins did not contain the "KAG" motif of unknown function expected of the cellulose synthase subfamily [28,29]. In replacement, *C. variabilis* and C-169 have a "KSG" or "KSA" motif.

**Figure S7**. Similarity tree of algal short-chain dehydrogenase protein domains

Proteins of sequenced chlorophytes that have significant match to the adh_short PFAM motif were aligned using MUSCLE. A neighbour joining tree was constructed from the multiple-sequence alignment using CLUSTALW and P distance, without removing gapped positions. This similarity tree is an approximation of the real phylogenetic tree. A robust phylogenetic tree could not be reconstructed using a standard procedure due to a high number of gapped positions in the multiple-alignment. Proteins are symbolized by shaded shapes to indicate to what species they belong to. See the color code caption in the figure for more details. Green branches highlight putative subfamilies that are specifically expanded in the C-169 lineage.

**Figure S8**: Multiple alignment of *Chlamydomonas reinhardtii* CIA5 with putative orthologs from C-169, *Chlorella variabilis* and *Volvox carteri*.

Only the N-terminal portion of alignments are shown. Residues critical for zinc binding and/or response to carbon-limited conditions in *C. reinhardtii* are marked (▼). The *Volvox carteri* protein sequence was acquired from Fukuzawa et al., 2001 .

**Figure S9**: Principal component analysis (PCA) of amino acid composition in plantae proteomes.

(A) Component scores for organisms; (B) component loadings for amino acids. PCA can be applied to a multivariate data set in order to reduce the complexity of the data and to determine whether there are underlying trends that explain the observed variation. The first two principal components (PCs) accounted for 78.3% of the variance in amino acid composition. PC 2 correlated strongly with genome GC content (correlation coefficients for PC1 and PC2 scores vs. GC content were 0.25 and -0.91, respectively). The influence of GC content on PCs 1 and 2 was apparent, with amino acids encoded by high GC codons (Gly, Ala, Arg, Val, Pro) appearing on the bottom right of the plot while amino acids encoded by low GC codons (Tyr, Lys, Phe, Ile, Asn) appearing on the top left of the plot.

The amino acid composition in proteins from C-169 is not sufficiently different to distinguish it from the mesophilic Plantae species. In fact, the amino acid compositions of Streptophytes (*P. patens, O. sativa, A. thaliana*), Chlorophyceae (*C. reinhardtii, V. carteri*) and Trebouxiophyceae (*C. variabilis, C. subellipsoidea*) appears to be mainly influenced by the genome GC content. In contrast, the presence of the unicellular red alga *Cyanidioschyzon merolae* as an outlier on top right side of the plot can be attributed to the overrepresentation of basic amino acids (Histidine and arginine), which may represent an adaptation to highly acidic environments. Furthermore marine *Ostreococcus* and *Micromonas* species are clustered in the bottom left side of the plot in particular because they have an

overrepresentation of acidic amino acids, which is a known adaptation of halophilic species [30].

## 5 Supporting References


1. Agarkova IV, Dunigan DD, Van Etten JL: **Virion-Associated Restriction Endonucleases of Chloroviruses**. *J. Virol.* 2006, **80**:8114–8123.

2. Higashiyama T, Yamada T: **Electrophoretic karyotyping and chromosomal gene mapping of Chlorella**. *Nucleic Acids Research* 1991, **19**:6191 –6195.

3. Huang X, Madan A: **CAP3: A DNA Sequence Assembly Program**. *Genome Research* 1999, **9**:868 –877.

4. Swofford D: *PAUP*. Phylogenetic Analysis Using Parsimony (*and Other Methods). Version 4.* Sunderland, Massachusetts: Sinauer Associates; 2003.

5. Posada D, Crandall KA: **MODELTEST: testing the model of DNA substitution.** *Bioinformatics* 1998, **14**:817 –818.

6. Holm-Hansen O: **Isolation and Culture of Terrestrial and Fresh-water Algae of Antarctica**. *Phycologia* 1964, **4**:43–51.

7. Luo W, Pflugmacher S, Pröschold T, Walz N, Krienitz L: **Genotype versus phenotype variability in Chlorella and Micractinium (Chlorophyta, Trebouxiophyceae)**. *Protist* 2006, **157**:315–333.

8. Zoller S, Lutzoni F: **Slow algae, fast fungi: exceptionally high nucleotide substitution rate differences between lichenized fungi Omphalina and their symbiotic green algae Coccomyxa**. *Molecular Phylogenetics and Evolution* 2003, **29**:629–640.

9. Coleman AW, Mai JC: **Ribosomal DNA ITS-1 and ITS-2 sequence comparisons as a tool for predicting genetic relatedness**. *J. Mol. Evol.* 1997, **45**:168–177.

10. Acton E: **Coccomyxa subellipsoidea, a new member of the Palmellaceae**. *Annals of Botany* 1909, **23**:573–578.

11. Jaag O: **Coccomyxa Schmidle, Monographie einer Algengattung. - Beitr. Kryptogamenfl.** *Schwiez* 1933, **8**:1–132.

12. Higashiyama T, Maki S, Yamada T: **Molecular organization of Chlorella vulgaris chromosome I: presence of telomeric repeats that are conserved in higher plants**. *Mgg Molecular & General Genetics* 1995, **246**:29–36.

13. Derelle E, Ferraz C, Rombauts S, Rouzé P, Worden AZ, Robbens S, Partensky F, Degroeve S, Echeynié S, Cooke R, Saeys Y, Wuyts J, Jabbari K, Bowler C, Panaud O, Piégu B, Ball SG, Ral J-P, Bouget F-Y, Piganeau G, De Baets B, Picard A, Delseny M, Demaille J, Van de Peer Y, Moreau H: **Genome analysis of the smallest free-living eukaryote Ostreococcus tauri unveils many unique features**. *Proceedings of the National Academy of Sciences* 2006, **103**:11647 –11652.

14. Matsuzaki M, Misumi O, Shin-i T, Maruyama S, Takahara M, Miyagishima S, Mori T, Nishida K, Yagisawa F, Nishida K, Yoshida Y, Nishimura Y, Nakao S, Kobayashi T, Momoyama Y, Higashiyama T, Minoda A, Sano M, Nomoto H, Oishi K, Hayashi H, Ohta F, Nishizaka S, Haga S, Miura S, Morishita T, Kabeya Y, Terasawa K, Suzuki Y, Ishii Y, et al.:

**Genome sequence of the ultrasmall unicellular red alga Cyanidioschyzon merolae 10D**. *Nature* 2004, **428**:653–657.

15. Merchant SS, Prochnik SE, Vallon O, Harris EH, Karpowicz SJ, Witman GB, Terry A, Salamov A, Fritz-Laylin LK, Maréchal-Drouard L, Marshall WF, Qu L-H, Nelson DR, Sanderfoot AA, Spalding MH, Kapitonov VV, Ren Q, Ferris P, Lindquist E, Shapiro H, Lucas SM, Grimwood J, Schmutz J, Cardol P, Cerutti H, Chanfreau G, Chen C-L, Cognat V, Croft MT, Dent R, et al.: **The Chlamydomonas Genome Reveals the Evolution of Key Animal and Plant Functions**. *Science* 2007, **318**:245 –250.

16. Blanc G, Duncan G, Agarkova I, Borodovsky M, Gurnon J, Kuo A, Lindquist E, Lucas S, Pangilinan J, Polle J, Salamov A, Terry A, Yamada T, Dunigan DD, Grigoriev IV, Claverie J-M, Van Etten JL: **The Chlorella variabilis NC64A Genome Reveals Adaptation to Photosymbiosis, Coevolution with Viruses, and Cryptic Sex**. *The Plant Cell* 2010, **22**:2943–2955.

17. Palenik B, Grimwood J, Aerts A, Rouzé P, Salamov A, Putnam N, Dupont C, Jorgensen R, Derelle E, Rombauts S, Zhou K, Otillar R, Merchant SS, Podell S, Gaasterland T, Napoli C, Gendler K, Manuell A, Tai V, Vallon O, Piganeau G, Jancek S, Heijde M, Jabbari K, Bowler C, Lohr M, Robbens S, Werner G, Dubchak I, Pazour GJ, et al.: **The tiny eukaryote Ostreococcus provides genomic insights into the paradox of plankton speciation**. *Proceedings of the National Academy of Sciences* 2007, **104**:7705 –7710.

18. Giordano M, Beardall J, Raven JA: **CO2 concentrating mechanisms in algae: mechanisms, environmental modulation, and evolution**. *Annu Rev Plant Biol* 2005, **56**:99–131.

19. Prochnik SE, Umen J, Nedelcu AM, Hallmann A, Miller SM, Nishii I, Ferris P, Kuo A, Mitros T, Fritz-Laylin LK, Hellsten U, Chapman J, Simakov O, Rensing SA, Terry A, Pangilinan J, Kapitonov V, Jurka J, Salamov A, Shapiro H, Schmutz J, Grimwood J, Lindquist E, Lucas S, Grigoriev IV, Schmitt R, Kirk D, Rokhsar DS: **Genomic analysis of organismal complexity in the multicellular green alga Volvox carteri**. *Science* 2010, **329**:223–226.

20. Worden AZ, Lee J-H, Mock T, Rouzé P, Simmons MP, Aerts AL, Allen AE, Cuvelier ML, Derelle E, Everett MV, Foulon E, Grimwood J, Gundlach H, Henrissat B, Napoli C, McDonald SM, Parker MS, Rombauts S, Salamov A, Von Dassow P, Badger JH, Coutinho PM, Demir E, Dubchak I, Gentemann C, Eikrem W, Gready JE, John U, Lanier W, Lindquist EA, et al.: **Green Evolution and Dynamic Adaptations Revealed by Genomes of the Marine Picoeukaryotes Micromonas**. *Science* 2009, **324**:268 –272.

21. Armbrust EV, Berges JA, Bowler C, Green BR, Martinez D, Putnam NH, Zhou S, Allen AE, Apt KE, Bechner M, Brzezinski MA, Chaal BK, Chiovitti A, Davis AK, Demarest MS, Detter JC, Glavina T, Goodstein D, Hadi MZ, Hellsten U, Hildebrand M, Jenkins BD, Jurka J, Kapitonov VV, Kröger N, Lau WWY, Lane TW, Larimer FW, Lippmeier JC, Lucas S, et al.: **The genome of the diatom Thalassiosira pseudonana: ecology, evolution, and metabolism**. *Science* 2004, **306**:79–86.

22. Gobler CJ, Berry DL, Dyhrman ST, Wilhelm SW, Salamov A, Lobanov AV, Zhang Y, Collier JL, Wurch LL, Kustka AB, Dill BD, Shah M, VerBerkmoes NC, Kuo A, Terry A, Pangilinan J, Lindquist EA, Lucas S, Paulsen IT, Hattenrath-Lehmann TK, Talmage SC,

Walker EA, Koch F, Burson AM, Marcoval MA, Tang Y-Z, Lecleir GR, Coyne KJ, Berg GM, Bertrand EM, et al.: **Niche of harmful alga Aureococcus anophagefferens revealed through ecogenomics**. *Proc. Natl. Acad. Sci. U.S.A.* 2011, **108**:4352–4357.

23. Bowler C, Allen AE, Badger JH, Grimwood J, Jabbari K, Kuo A, Maheswari U, Martens C, Maumus F, Otillar RP, Rayko E, Salamov A, Vandepoele K, Beszteri B, Gruber A, Heijde M, Katinka M, Mock T, Valentin K, Verret F, Berges JA, Brownlee C, Cadoret J-P, Chiovitti A, Choi CJ, Coesel S, De Martino A, Detter JC, Durkin C, Falciatore A, et al.: **The Phaeodactylum genome reveals the evolutionary history of diatom genomes**. *Nature* 2008, **456**:239–244.

24. Fukuzawa H, Miura K, Ishizaki K, Kucho KI, Saito T, Kohinata T, Ohyama K: **Ccm1, a regulatory gene controlling the induction of a carbon-concentrating mechanism in Chlamydomonas reinhardtii by sensing CO2 availability**. *Proc. Natl. Acad. Sci. U.S.A.* 2001, **98**:5347–5352.

25. Xiang Y, Zhang J, Weeks DP: **The Cia5 gene controls formation of the carbon concentrating mechanism in Chlamydomonas reinhardtii**. *Proc. Natl. Acad. Sci. U.S.A.* 2001, **98**:5341–5346.

26. Yamano T, Fujita A, Fukuzawa H: **Photosynthetic characteristics of a multicellular green alga Volvox carteri in response to external CO(2) levels possibly regulated by CCM1/CIA5 ortholog**. *Photosyn. Res.* 2011, **109**:151–159.

27. Charnock SJ, Henrissat B, Davies GJ: **Three-dimensional structures of UDP-sugar glycosyltransferases illuminate the biosynthesis of plant polysaccharides**. *Plant Physiol.* 2001, **125**:527–531.

28. Saxena IM, Brown RM Jr: **Cellulose synthases and related enzymes**. *Curr. Opin. Plant Biol.* 2000, **3**:523–531.

29. Richmond TA, Somerville CR: **The Cellulose Synthase Superfamily**. *Plant Physiology* 2000, **124**:495 –498.

30. Kennedy SP, Ng WV, Salzberg SL, Hood L, DasSarma S: **Understanding the adaptation of Halobacterium species NRC-1 to its extreme environment through computational analysis of its genome sequence**. *Genome Res.* 2001, **11**:1641–1650.
